# EIF4a3-regulated circRABL2B regulates cell stemness and drug sensitivity of lung cancer via YBX1-dependent downregulation of MUC5AC expression

**DOI:** 10.7150/ijbs.78588

**Published:** 2023-05-21

**Authors:** Liming Lu, Yuyuan Zeng, Ziqi Yu, Shizhen Chen, Jianjiang Xie, Boqi Rao, Binyao Yang, Fuman Qiu, Jiachun Lu, Lei Yang

**Affiliations:** 1 The State Key Lab of Respiratory Disease, Institute of Public Health, Guangzhou Medical University, Guangzhou 511436, China.; 2 Department of Pulmonary and Critical Care Medicine, Guangzhou First People's Hospital, the Second Affiliated Hospital of South China University of Technology, Guangzhou 510080, China.; 3 Innovation center for Advanced Interdisciplinary Medicine, The Fifth Affiliated Hospital of Guangzhou Medical University, Guangzhou 510735, China.; 4 The State Key Lab of Respiratory Disease, Guangzhou Institute of Respiratory Diseases, The First Affiliated Hospital of Guangzhou Medical University, Guangzhou Medical University, Guangzhou 510120, China.

**Keywords:** mucins, circRABL2B, lung cancer, exosomes

## Abstract

Identification of mucin modulators is of remarkable significance to facilitate mucin-based antineoplastic therapy. However, little is known about circular RNAs (circRNAs) on regulating mucins. Dysregulated mucins and circRNAs were identified via high-throughput sequencing and their relationships with lung cancer survival were analyzed in tumor samples of 141 patients. The biological functions of circRABL2B were determined via gain- and loss-of-function experiments and exosome-packaged circRABL2B treatment in cells, patient-derived lung cancer organoids and nude mice. We identified that circRABL2B was negatively correlated with MUC5AC. Patients with low circRABL2B and high MUC5AC displayed the poorest survival (HR=2.00; 95% CI=1.12-3.57). Overexpressed circRABL2B significantly inhibited cell malignant phenotypes, while it knock-down exerted opposite effects. CircRABL2B interacted with YBX1 to inhibit MUC5AC, and subsequently suppressed integrin β4/pSrc/p53 signaling and impoverished cell stemness, and promoted erlotinib sensitivity. Exosome-packaged circRABL2B exerted significant anti-cancer actions in cells, patient-derived lung cancer organoids and nude mice. Meanwhile, circRABL2B in plasma exosomes could distinguish early-stage lung cancer patients from healthy controls. Finally, we found circRABL2B was downregulated at the transcriptional level, and EIF4a3 involved the formation of circRABL2B. In conclusion, our data suggest that circRABL2B counteracts lung cancer progression via MUC5AC/integrin β4/pSrc/p53 axis, which provides a rationale to enhance the efficacy of anti-MUCs treatment in lung cancer.

## Background

Lung cancer remains the leading cause of cancer-related death^1^. In the past decade, significant advances have been achieved in the treatment of lung cancer. Molecular therapies (MT), such as immune checkpoint blockade therapies (ICBs), have shown incremental benefits in treating advanced lung cancer, but tumor heterogeneity and evolution pose long-lasting challenges as that many patients respond poorly to MT and develop resistance^2^. Therefore, the development of novel MT drugs has been applied to overcome the problem. Mucins (MUCs) are a family of high molecular weight, heavily glycosylated proteins produced by epithelial cells, which have been widely reported to be associated with the progression of various cancers and were recently identified as new targets of ICBs^3^-^5^. On the ClinicalTrials Website (https://www.clinicaltrials.gov/), a total of 157 studies were found for anti-MUC agents in cancer. However, the upstream regulatory of MUCs remains unclear. Considering the challenges of MT, we have to understand the molecular mechanism underlying MUCs regulation, which will benefit anti-MUC therapy in future.

Circular RNA (circRNA) is a novel class of non-coding RNA with a closed-loop structure. Emerging evidence shows that circRNAs play essential roles in physiological processes and development of human diseases^6^, ^7^. Researches have so far identified many circRNAs to be involved in lung cancer tumorigenesis and progression^8^, ^9^. For instance, circNDUFB2 inhibits lung cancer progression by functioning as a scaffold to enhance interaction between TRIM25 and IGF2BPs^10^. Regulatory circRNAs on MUCs have been finitely studied in breast cancer, hepatocellular carcinoma, and ovarian cancer^11^-^14^, but their roles in lung cancer remains largely unknown.

In the present study, we identified a circRNA derived from RABL2B, hsa_circ_ 0064019 designated as circRABL2B, harbors a regulatory role in MUC5AC expression. CircRABL2B is downregulated in lung cancer tissues and is associated with poor survival. Mechanically, circRABL2B retards lung cancer progression by synergizing YBX1 to degrade MUC5AC mRNA and impoverish cancer cell stemness. Exosome-packaged high level of circRABL2B functions to inhibit lung cancer development, and its level in serum has a remarkably diagnostic value on early-stage lung cancer. Our work identified a novel tumor suppressor for lung cancer that is a promising new MT target for anti-MUCs therapy.

## Methods

### Patients and tissue specimens

A total of 141 histopathologically-confirmed lung cancer patients, who were successfully followed up semiannually by telephone were analyzed as described previously^15^. Lung cancerous tissues and corresponding noncancerous tissues were collected. Survival time was calculated from the date when patients were first diagnosed to the date of the last follow-up if survival or death day. Detailed information about the clinical characters of patients are listed in **Table S1**. This study was approved by the institutional review board at Guangzhou Medical University, and a written informed consent was obtained from each patient prior to this study.

### Expression microarray

Eight pairs of previously used lung cancer tissues and corresponding noncancerous were submitted to the Arraystar Human circRNA Array V2 analysis (8x15K, Arraystar Inc, Rockville, MD) to identify the expressional profile of circRNAs. Moreover, gene expressional profiles in response to circRABL2B overexpression or knockdown were determined using Agilent SurePrint G3 Human Gene Expression v2.0 Microarray (8 × 60K) at SINOTECH GENOMICS Company (Shanghai, China). Kyoto encyclopedia of genes (KEGG) and gene set enrichment analysis (GSEA) were performed using R software and GSEA 4.1.0 with default parameters.

### Isolation of side population cells and tumor spheroid assay

Side population (SP) cells of A549 or PC9 were analyzed and sorted using the method described by Maria M. Ho^16^. Briefly, 1 × 10^6^ cells were labeled with 5 µg/ml Hoechst33342 (Thermo Fisher Scientific) alone or in combination with 50 µg/ml verapamil (MedChemExpress, Monmouth Junction, NJ). Then the cells were centrifuged and suspended in a culture medium containing 2% FBS, and incubated with 1 μg/ml propidium iodide (Thermo Fisher Scientific). SP analysis and sorting were done on MoFlo XDP cell sorter (Beckman, Brea, CA). Isolated SP cells were seeded into 24-well Ultra Low Cluster plates (Corning) and cultured in a complete culture medium (ThermoFisher Scientific) containing DMEM/F12 (1:1), EGF (20 ng/mL), bFGF (20 ng/mL), and B27 (2%). After 10-14 days, spheres were counted under an inverse microscope.

### Generation of circRABL2B-overexpressed lung cancer organoid

Two lung cancer organoids (LCOs) were produced from tumor surgical resections of two lung cancer patients using the Accuroid™ lung cancer organoid Kit, according to the manual (ACCURATE International. Guangzhou, China). Among them, one LCO was randomly selected for circRABL2B lentiviral transfection, as described^17^. Briefly, the LCO was dissociated, precipitated and re-suspended in 250 ul lentiviral solution. Spinoculation was executed by centrifuging the culture plate at 600g for one hour at 32ºC. The mixture was then incubated at 37ºC for 6 hours. After removing the infection medium, the cells were pelleted to be embedded in fresh Matrigel media mixture by hanging drop method. Stable LCO cultures were selected by antibiotics.

### Exosome quantification, staining and treatment

For quantification, exosomes pellets were treated with Exosome ELISA Complete Kits (System Biosciences) and quantitated using ELISA assay following the operating manual. For staining, exosomes were treated with DiO or DiR Labeling Dye (Vybrant Multi-Color Cell Labeling Kit; Life Technologies) for 20 min. The DiO-labeled exosomes were incubated with recipient cells, which was followed by a routine immunofluorescence (IF) staining. The DiR-labeled exosomes were injected intratumorally. For *in vitro* exosomes treatment, approximately 9 × 10^9^ exosomes in 100 μl PBS were added to recipient cells, which was followed by *in vitro* cell behavior assays after 24-48 h. For *in vivo* exosomes treatment, 9 × 10^10^ exosomes were injected intratumorally every other day for three times, when the xenografts in nude mice grew to a visibly similar size. For *ex vivo* exosomes treatment, approximately 9 × 10^9^ exosomes were added to the LCOs, and live/dead discrimination of cells was measured using LIVE/DEAD Viability/Cytotoxicity Kit (Thermo Fisher).

### Other experiments

The protocols for cell culture, gene expression test, Ribonuclease R treatment, vector construction and cell transfection, *in vitro* and *in vivo* cell behavior assays, IF staining, RNA fluorescence in situ hybridization (FISH), RNA pull-down, RNA immunoprecipitation (RIP), and chromatin immunoprecipitation (ChIP), exosome isolation and identification, are described in **Supplementary materials**. Information about primers, probes and antibodies are listed in **Table S2** and** S3**.

### Statistical analysis

Differences between paired data were analyzed using the Wilcoxon matched-pairs signed rank test, and those between the measurement data were tested using Student's *t* test or Welch's t test. Analysis of repeated measurement data was performed using the Two-way ANOVA test. Correlations between indicated circRNAs and MUCs were analyzed with the Spearman correlation test. Survival analysis was performed using the log-rank test and Cox regression model. All the tests were performed using the GraphPad Prism 9.0.1 software (CA, USA). Moreover, the best cut-off point of prognosis with regarding to levels of circRABL2B or MUC5AC was determined using the Cox regression model in the “survminer” package of R 4.0.1. Two-side *P* < 0.05 was considered to be statistically significant.

## Results

### CircRABL2B is downregulated in lung cancer and negatively correlated with MUC5AC

**Figure 1:**
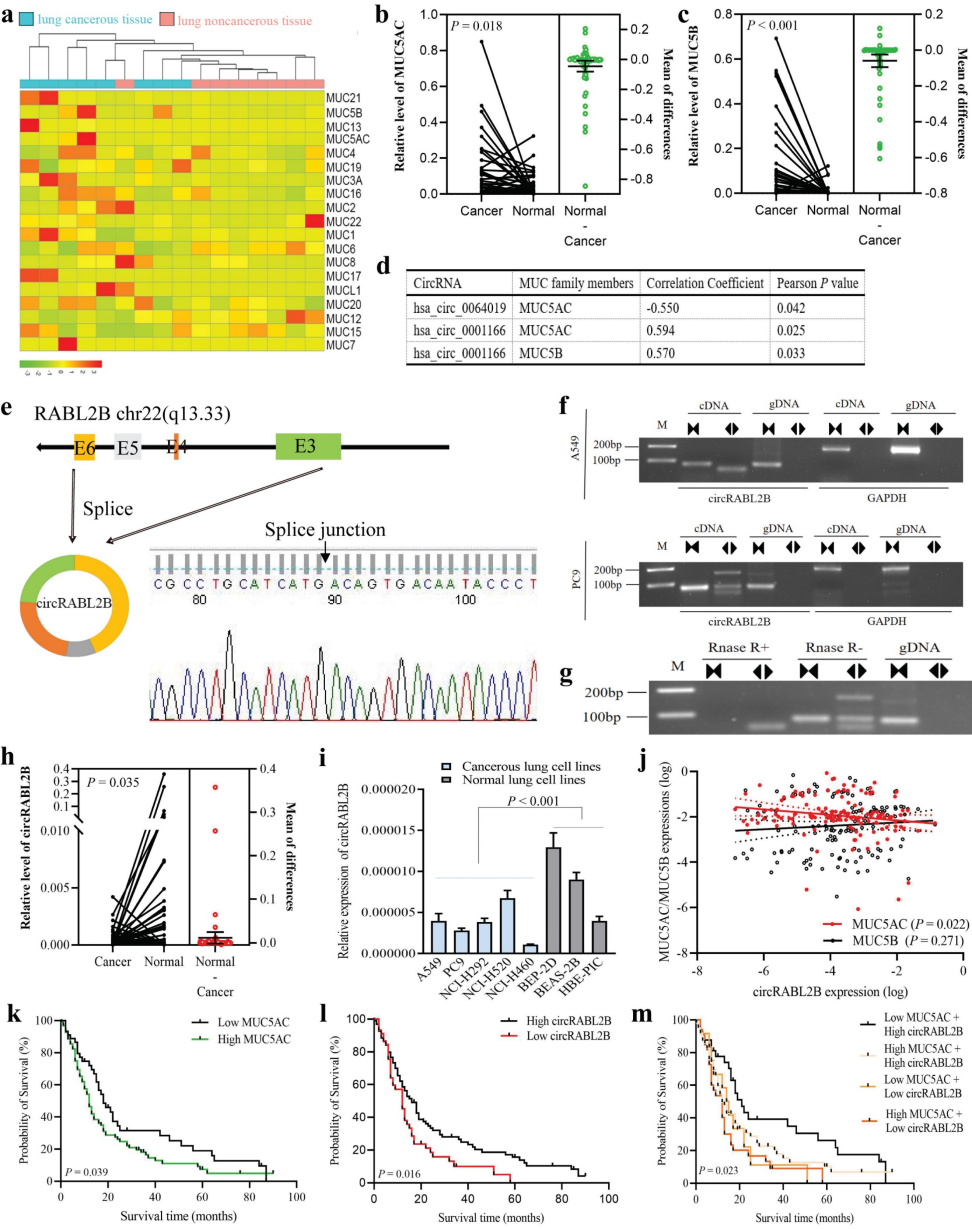
** circRABL2B is negatively correlated with MUC5AC and associated with lung cancer survival. a** A heatmap plot shows expressional profile of MUCs in eight paired samples of lung cancer by RNA-seq. Cut off is |Log_2_(fold change)|>1.5 and* P* value < 0.05. **b-c** Analysis for mRNA levels of MUC5AC (b) and MUC5B (c) in additional 68 paired samples of lung cancer. Cancer: cancerous lung tissue, Normal: corresponding noncancerous lung tissue. **d** circRNAs with significant correlations with MUC5AC or MUC5B, as revealed by the co-expressional analysis. **e-g** Schematic representation and characterization of circRABL2B formation. The backsplice junction site of circRABL2B was identified by Sanger sequencing (e). PCR amplification of circRABL2B and GAPDH with complementary DNA (cDNA) or genomic DNA (gDNA) in A549 and PC9 (f). PCR analysis for circRABL2B after treatment with or without RNase (g). **h** Analysis for circRABL2B levels in 68 paired samples of lung cancer. The differences between cancer and normal are presented as Mean ± S.E. in b, c and h. *P* values are calculated by Wilcoxon matched-pairs signed rank test in b, c and h. **i** qPCR analysis for circRABL2B expression in lung cell lines. Data are presented as Mean ± S.E. The *P* value is tested by student's* t* test. n = 6-7 biologically independent samples for each cell line. **j** Correlations between expressions of MUC5AC, MUC5B and circRABL2B. *P* values are calculated by Spearman correlation test. **k-m** Kaplan-Meier survival curves for lung cancer patients according to expressions of MUC5AC (k), circRABL2B (l) and their combinations (m). *P* values are calculated by Log-rank test.

We analyzed our previously-published RNA-seq data on eight pairs of lung cancer tissues and adjacent normal tissues^18^, and found three MUCs that are MUC5AC, MUC5B, and MUC3A were significantly up-regulated in lung cancer (**Fig. 1a**). By determining expressions of the three MUCs in additional 68 pairs of samples, we confirmed the upregulations of MUC5AC (**Fig. 1b**) and MUC5B (**Fig. 1c**) but not MUC3A (**Fig. S1a**) in cancer tissues. We then performed a co-expressional analysis between the abnormally expressed circRNAs, as revealed by the circRNA array, and MUC5AC, MUC5B, and found that circRABL2B, was negatively correlated with MUC5AC (*r* = -0.550, *P* = 0.042; **Fig. 1d**), and hsa_circ_0001166 that derives from *SULF2*, designated as circSULF2, was positively correlated with MUC5AC (*r* = 0.594, *P* = 0.025) and MUC5B (*r* = 0.570, *P* = 0.033). We further confirmed that circRABL2B is a bona fide circRNA that is generated from the exons 3-6 of* RABL2B* and harbors a closed-loop structure (**Fig. 1e-g**). The sequence of circRABL2B is consistent with circBase database annotation (http://www.circbase.org/). In additional, same analyses showed that circSULF2 is also a bona fide circRNA that derives from the exon 11 of *SULF2* (**Fig. S1b-d**).

The circRABL2B is downregulated in lung cancer tissues in comparison to adjacent normal lung tissues (**Fig. 1h**), and in lung cancer cell lines when compared to normal lung cell lines (**Fig. 1i**). In the cohort of 141 lung cancer patients, we confirmed the negative correlation of expressions between circRABL2B and MUC5AC (*r* = -0.193,* P* = 0.022; **Fig. 1j**). Meanwhile, we found that high level of MUC5AC (*P* = 0.039; **Fig. 1k**), low expression of circRABL2B (*P* = 0.016; **Fig. 1l**) were associated with poor survival of lung cancer patients. Besides, patients with both high MUC5AC and low circRABL2B exerted the worst survival after adjustment for age, sex, smoking status, family history of lung cancer, and clinical stage (HR=2.00; 95%CI=1.12-3.57; **Fig. 1m, Table 1**). Being consistent, data from the Cancer Genome Atlas (TCGA) database also showed that high MUC5AC level indicated poor survival (http://kmplot.com/analysis/index.php?p=service&cancer=lung; **Fig. S1e**). In addition, the circSULF2 is up-regulated in lung cancer tissues in comparison with adjacent normal lung tissues (**Fig. S1f**), and in lung cancer cell lines when compared to normal lung cell lines (**Fig. S1g**), and its expression is positively correlated with MUC5B (*r* = 0.266,* P* = 0.001) but not MUC5AC (**Fig. S1h**). However, MUC5B and circSULF2 levels were not associated with survival (**Fig. S1i, j**). The above results suggest that circRABL2B is correlated with MUC5AC and their dysregulated expressions are associated with the survival of lung cancer.

### CircRABL2B functions as tumor suppressor in lung cancer

**Figure 2:**
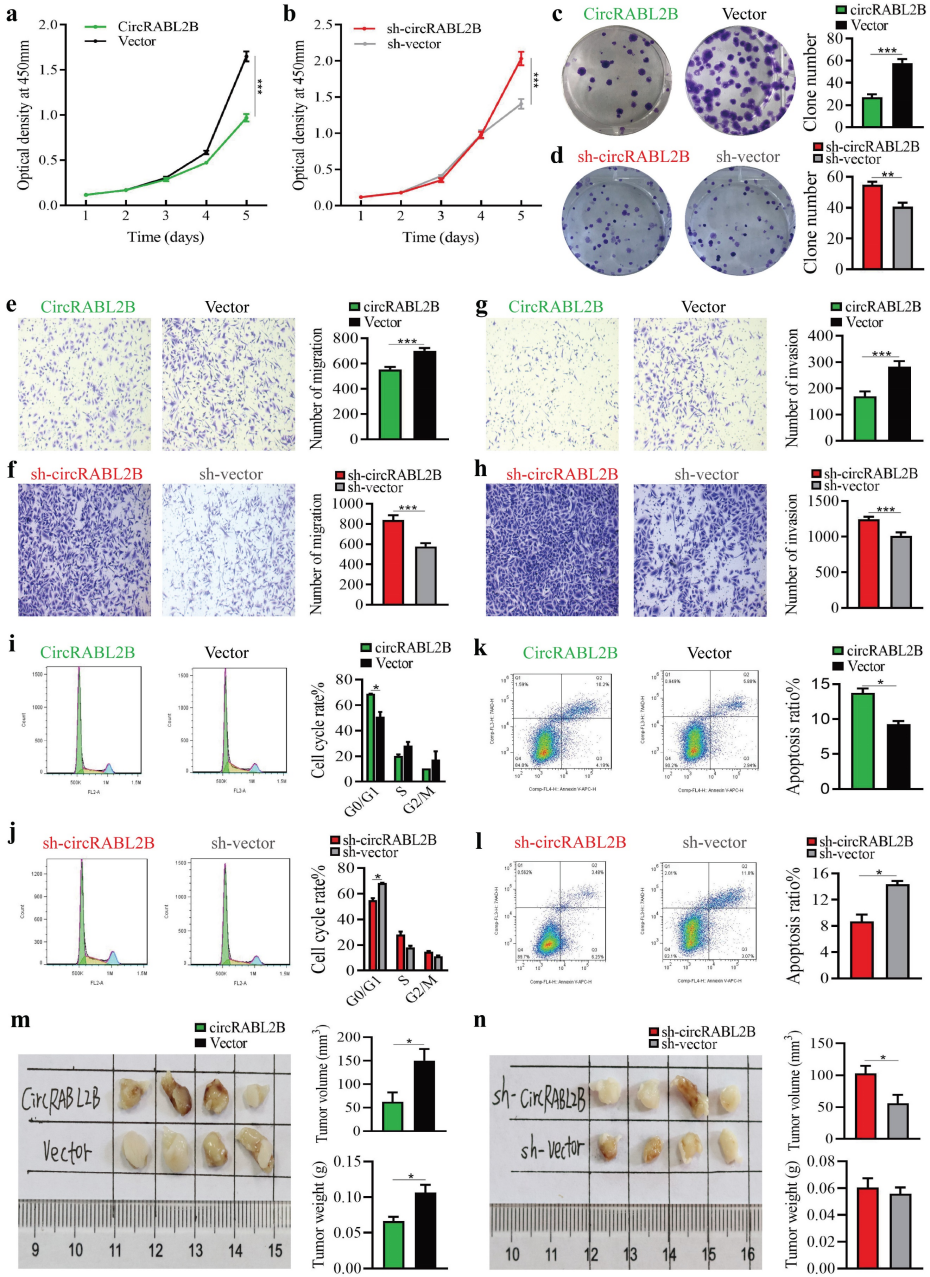
** CircRABL2B inhibits proliferation and metastasis of A549 *in vitro* and *in vivo*. a-l** Assays for testing cell proliferation (a, b), colony formation (c, d), migration ( e, f) and invasion (g, h), for exanimating cell cycle (i, j) and apoptosis (k, l) in A549 cells with circRABL2B overexpression (circRABL2B) versus vector control (vector), or cells with circRABL2B knock-down (sh-circRABL2B) versus shRNA vector control (sh-vector). n = 3-6 biologically independent samples for different assays. **m, n.** *In vivo* tumorigenesis assays for A549 with or without circRABL2B overexpression or PC9 with or without circRABL2B knock-down (n = 4 mice per group). Data are presented as Mean ± S.E. and *P* values are calculated by Two-way ANOVA test in a, b and unpaired *t* test in c-n. * *P* < 0.05, ** *P* < 0.01, *** *P* < 0.001.

We constructed circRABL2B overexpression plasmid and shRNA plasmid, and confirmed circRABL2B was steadily overexpressed or inhibited in lung cancer cells (**Fig. S2a, b**). Overexpressed circRABL2B repressed cell proliferation, clone formation, migration, invasion, and division (**Fig 2a, c, e, g, i, Fig. S2c, e, g, i, k**), while circRABL2B inhibition promoted these phenotypes (**Fig. 2b, d, f, h, j; Fig. S2d, f, h, j, l**). Meanwhile, overexpressed circRABL2B facilitated cell apoptosis (**Fig. 2k, Fig. S2m),** and knockdown of it exerted an opposite effect (**Fig. 2l, Fig. S2n**). Furthermore, circRABL2B overexpression decelerated tumor growth of lung cancer *in vivo* (**Fig. 2m**). Reverse effects were observed when silencing circRABL2B but were not as evident as overexpressed circRABL2B (**Fig. 2n**). However, shRNA-mediated knock-down of circSULF2 did not significantly affect the above phenotypes (**Fig. S3**). Thus, circSULF2 was not further studied. These data support that circRABL2B functions as a tumor suppressor in lung cancer.

### CircRABL2B interacts with YBX1 to suppress MUC5AC expression

**Figure 3:**
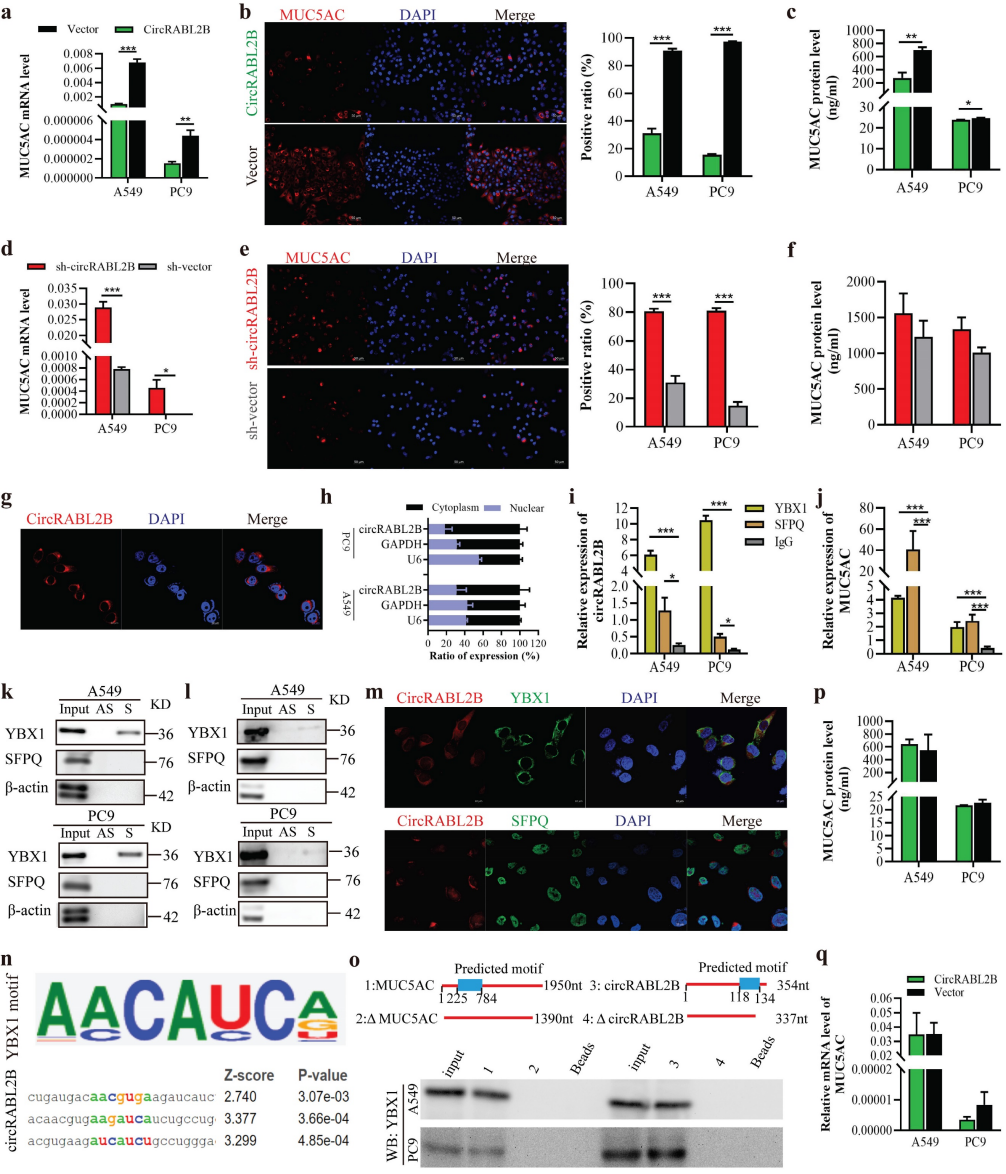
** CircRABL2B physically interacts with YBX1 to suppress MUC5AC expression. a-f** Determination of MUC5AC expression at levels of mRNA by qPCR (a, d), protein by IF staining (b, e; Scale bar = 50 μm) and ELISA (c, f), in lung cancer cells with circRABL2B overexpression (circRABL2B) versus vector control (vector), or in cells with circRABL2B knock-down (sh-circRABL2B) versus shRNA vector control (sh-vector). **g** RNA FISH for circRABL2B subcellular localization in lung cancer cells. Scale bar = 10 μm. **h** Expression levels of circRABL2B, GAPDH, and U6 in the nuclear and cytoplasmic fractions of A549 and PC9. **i, j** RIP assays for analyzing binding abilities of YBX1 and SFPQ on circRABL2B (i) or MUC5AC transcript (j) in lung cancer cells. **k, l** RNA pull-down assays for detecting interactions between YBX1, SFPQ and circRABL2B (k) as well as MUC5AC (l) in lung cancer cells. Two independent experiments were carried out with similar results. **m** Co-localization of circRABL2B (red) with YBX1 or SFPQ (green) in lung cancer cells. Scale bar = 10 μm. **n** *In silico* analysis for binding site visualization at circRABL2B for YBX1. **o** Truncation mapping of circRABL2B-YBX1 binding domain, and MUC5AC-YBX1 binding domain. Top panel: diagrams of 5'-end of MUC5AC, circRABL2B full length and truncated fragments. Bottom: WB of YBX1 pulled down by different 5'-end of MUC5AC and circRABL2B fragments. **p, q** Levels of MUC5AC protein (p) and mRNA (q) in circRABL2B overexpressed lung cancer cells after YBX1 knock-down. Data are presented as Mean ± S.E from 3-6 biologically replicates. *P* values are calculated by unpaired *t* test. * *P* < 0.05, ** *P* < 0.01, *** *P* < 0.001.

The assays of qPCR, IF and ELISA uniformly showed that circRABL2B decreased MUC5AC expression, while it knock-down exerted opposite effects (**Fig. 3a-f**). Since circRABL2B is predominantly expressed in cytoplasm (**Fig. 3g, h**), we hypothesized that circRABL2B regulates MUC5AC expression at post-transcription level. RNA pull-down followed by mass spectrometry revealed a total of 238 RBPs with potentials to capture circRABL2B, which were enriched in several pathways, such as the EGFR signaling pathway as the KEGG analysis shown (**Fig. S4a, b**). These RBPs did not include AGO2, which means that circRABL2B may not act as a miRNA sponge. After blasting the 238 RBPs with 17 predicted RBPs who harbor binding sites at MUC5AC mRNA, as revealed by the catRAPID omics (http://service.tartaglialab.com/page/catrapid_group), two overlapped proteins were discovered: YBX1 and SFPQ (**Fig. S4c-e**). The RIP assay revealed that both YBX1 and SFPQ harbor a potential binding to circRABL2B (**Fig. 3i**) and MUC5AC mRNA (**Fig. 3j**), but the RNA pull-down showed only YBX1 physically interacted with circRABL2B (**Fig. 3k**) or MUC5AC transcript (**Fig. 3l**). Co-localization of circRABL2B with YBX1 but not SFPQ was also observed (**Fig. 3m**). *In silico* analysis (RBPmap: http://rbpmap.technion.ac.il/index.html) revealed potential binding sequences at circRABL2B and 5'-end of MUC5AC for YBX1 (**Fig. 3n, Fig. S4f**), we thus constructed truncated circRABL2B and MUC5AC with deletion of predicted motifs, and found that the sequences of circRABL2B (nucleotides 118-134), and MUC5AC (nucleotides 225-784) were required for their interactions with YBX1 (Fig.3o). Knock-down of YBX1 also significantly increased MUC5AC expression (**Fig. S5a, b**) and rendered MUC5AC to refrain from circRABL2B suppression (**Fig. 3p, q**), with a restoration of cell growth, migration and invasion caused by circRABL2B overexpression (**Fig. S5c-f**). The TCGA data consistently revealed that YBX1 expression was negatively correlated with MUC5AC level in lung cancer tissues (http://gepia.cancer-pku.cn/; **Fig. S5g**). In addition, circRABL2B did not affect both YBX1 and SFPQ expressions (**Fig. S5h**). Taken together, our data suggest circRABL2B functions to interact with YBX1 to regulate MUC5AC expression.

### Knock-down of MUC5AC rescues the biological effects of circRABL2B inhibition

To delineate whether MUC5AC is an essential target of circRABL2B on affecting malignant phenotypes of lung cancer, we knocked down MUC5AC by siRNAs (**Fig. S6a**) in circRABL2B knock-down cells, where MUC5AC expression was increased. Knock-down of MUC5AC significantly inhibited cell proliferation and migration of lung cancer cells (**Fig. S6b, c**). Moreover, MUC5AC knock-down prevented the circRABL2B shRNA-induced promotion of cell proliferation, colony formation, migration and invasion (**Fig. S6d-g**). These data suggest that circRABL2B works majorly through MUC5AC.

### CircRABL2B impoverishes lung cancer cell stemness via pSrc-integrin β4-p53 axis

**Figure 4:**
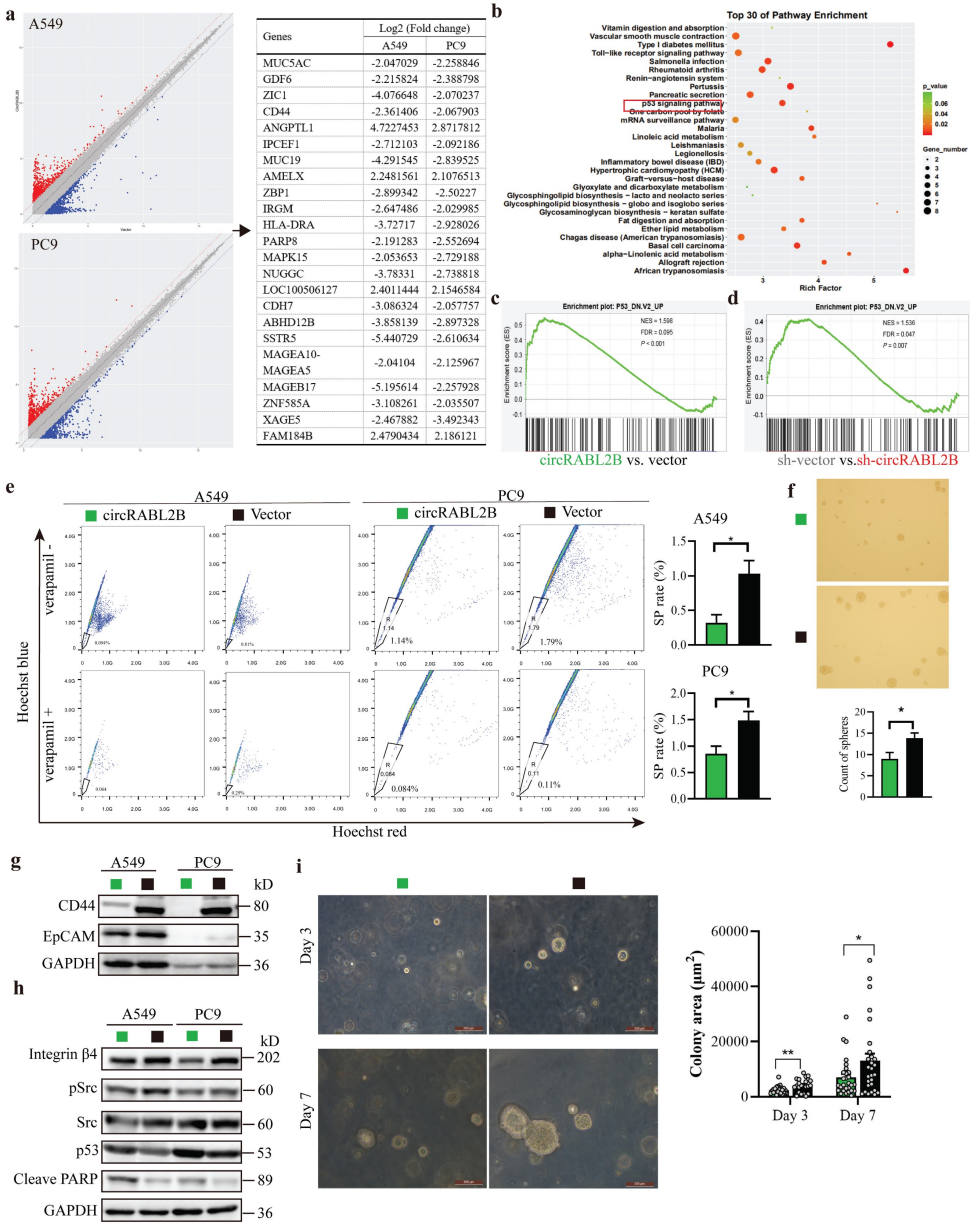
** CircRABL2B impoverishes lung cancer cell stemness via affecting pSrc-integrin β4-p53 axis. a** Gene microarray identified a large number of target genes in A549 and PC9 with circRABL2B overexpression versus vector control. **b** Enrichment analysis for top of 30 KEGG pathways in circRABL2B targets. **c** Gene set enrichment profile showing enrichment of the P53 signaling pathway for the circRABL2B overexpression group (circRABL2B) compared to the control group (vector). **d** Gene set enrichment profile showing enrichment of the P53 signaling pathway for the circRABL2B knock-down group (sh-circRABL2B) compared to the control group (sh-vector). NES normalized enrichment score, FDR false discovery rate. *P* values are calculated by permutation test. **e** Representative images and quantitative representation of percentage SP from SP analysis in A549 and PC9 with or without circRABL2B overexpression. n = 3 biologically independent samples for each cell line. **f** Representative micrographs showing the spheres by SP with or without circRABL2B overexpression. **g** WB showing a decrease in biomarkers of cancer stem cells in A549 and PC9 cells after circRABL2B overexpression. **h** WB showing circRABL2B disrupts integrin β4/pSrc/p53 axis. Two independent experiments were carried out with similar results in g and h. **i** circRABL2B overexpression by lentiviral transduction suppressed patient-derived LCOs at day 3 and day 7. Scale bar = 200 μm. Data are presented as Mean ± S.E. *P* values are calculated by unpaired *t* test in e, f, and i.

Hundreds of genes displayed a more than twofold change in expression after circRABL2B overexpression in A549 and PC9, of which 23 genes, including MUC5AC, were overlapped with congruent changes (**Fig. 4a**). KEGG and GSEA showed that these genes were enriched in p53 signaling (**Fig. 4b, c**). Genes affected by silencing circRABL2B confirmed the regulation of P53 signaling (**Fig. 4d, Fig. S7a, b**). Recently, it has been reported that suppressing MUC5AC impoverishes cancer cell stemness via interacting with CD44 and affecting pSrc-integrin β4-p53 axis in colorectal cancer^19^, we wondered whether circRABL2B recapitulated such effect in lung cancer. SP analysis showed that overexpressed circRABL2B reduced the percentage of SP cells from 1.03% to 0.32% in A549 cells and from 1.48% to 0.86% in PC9 cells (**Fig. 4e**). Since one-time isolation of SP cells from either A549 or PC9 did not generate spheres, we mixed SP cells from A549 and PC9 and found that overexpressed circRABL2B significantly reduced the number of spheres (**Fig. 4f**). Consistently, the biomarkers of cancer stem cells - CD44 and EpCAM - were both downregulated in circRABL2B overexpressed cells (**Fig. 4g**). CircRABL2B overexpression also reduced integrin-β4 and pSrc expressions (**Fig. 4h**), and increased p53 expression and an apoptotic marker - cleaved PARP. Knock-down of YBX1 also restored expressions of above proteins caused by circRABL2B overexpression (**Fig. S7c**). Since organoid culture has been demonstrated to be an *ex vivo* analysis of stem cell behavior, we overexpressed circRABL2B in one case of LCO that were derived from a lung cancer patient using lentiviral infection. After establishing the overexpression of circRABL2B in infected LCOs, we found that circRABL2B significantly suppressed LCOs formation with smaller area in comparison with the vector control at day 3 and day 7 (**Fig. 4i**). These findings suggested that circRABL2B could recapitulate the effect of MUC5AC inhibition on affecting pSrc-integrin β4-p53 axis and impoverish cancer cell stemness.

### Effect of circRABL2B on the erlotinib sensitivity

**Figure 5:**
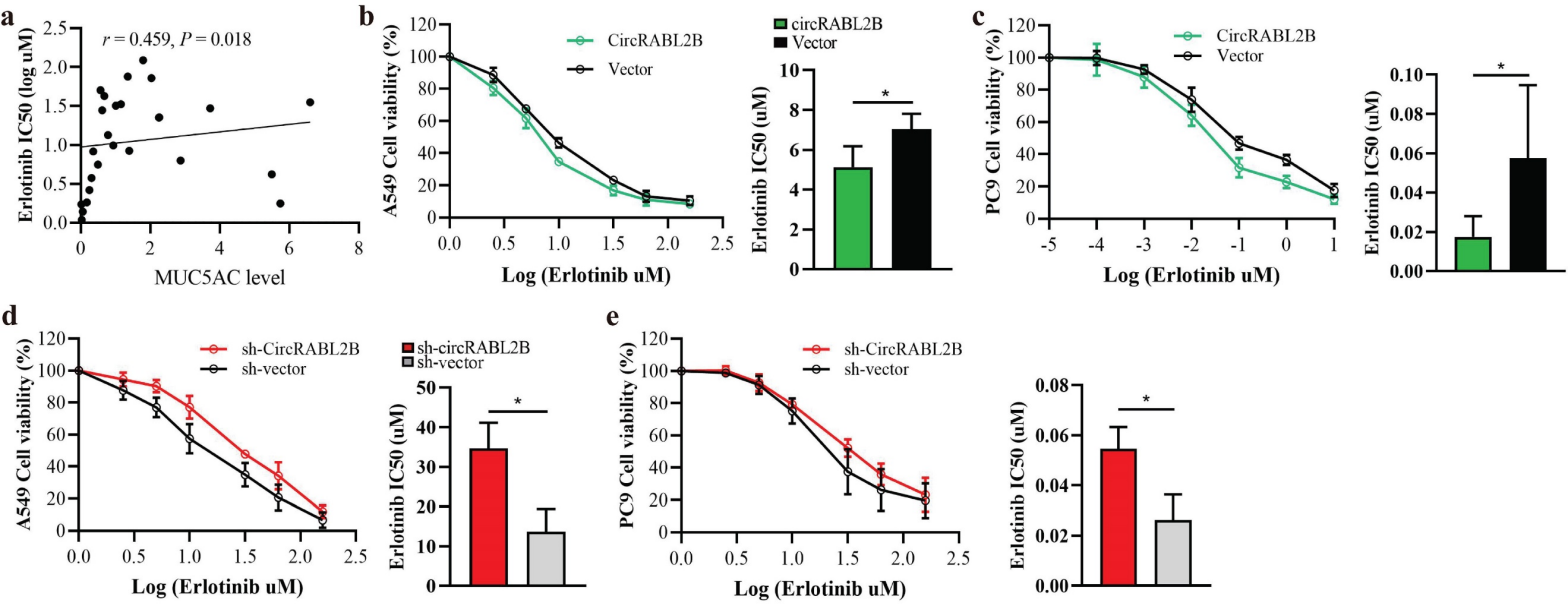
** Overexpression of circRABL2B promotes erlotinib sensitivity. a** *In silico* analysis revealing a positive correlation between MUC5AC and erlotinib resistance in lung cancer cells. **b, c** The CCK-8 assay showing that overexpression of circRABL2B promotes erlotinib sensitivity in A549 (b) and PC9 (c). **d, e** CircRABL2B silence accelerates erlotinib resistance. Data are presented as Mean ± S.D. from 3-5 biologically replicates. *P* values are calculated by Spearman rank correlation test in a, and unpaired *t* test in b-f.

To test whether circRABL2B affects drug sensitivity of lung cancer, we first searched the ClinicalTrials Website (https://www.clinicaltrials.gov/) to identify anti-MUC agents in clinical trials of lung cancer. We then obtained the expression of MUC5AC from the Cancer Cell Line Encyclopedia database (https://sites.broadinstitute.org/ccle/) and IC50 values of anti-MUC agents in various lung cancer cells from the Genomics of Drug Sensitivity in Cancer database (https://www.cancerrxgene.org/). The results showed that MUC5AC expression was positively correlated with the resistance of lung cancer cells to erlotinib (*r* = 0.459,* P* = 0.018; **Fig.5a**). We thus further tested whether circRABL2B affects the resistance of erlotinib using the CCK-8 cytotoxicity test. After overexpressing circRABL2B, both A549 and PC9 cells exhibited a decrease in drug resistance (**Fig.5b, c**), while after circRABL2B was silenced, both cells exerted an increase in drug resistance (**Fig.5d, e**).

### Exosome-packaged circRABL2B has a potential for treating lung cancer

**Figure 6:**
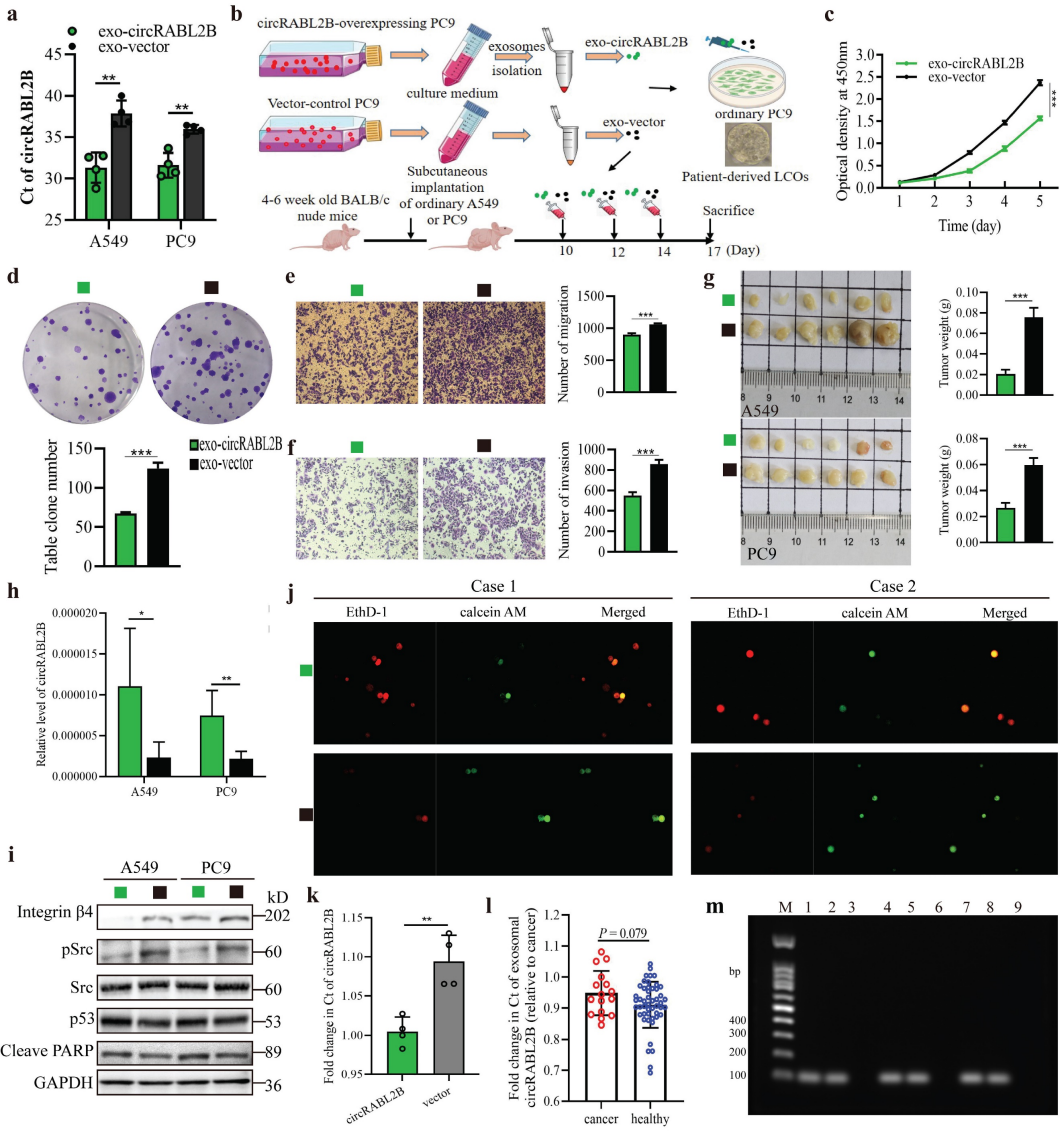
** Exosome-packaged circRABL2B has a potential for treating and diagnosing lung cancer. a** qPCR quantification of circRABL2B in exosomes from tissue culture medium. **b** Protocol of exosomes collection from culture medium and administration to ordinary PC9 cells, patient-derived LCOs, and nude mice. **c-f** Cell proliferation (c), colony formation (d), migration (e) and invasion (f) for PC9 cells after exo-circRABL2B or exo-vector treatment.** g** Tumor growth *in vivo* after exo-circRABL2B or exo-vector treatment. **h** qPCR quantification of circRABL2B in xenograft tumors after exo-circRABL2B or exo-vector treatment.** i** WB showing exo-circRABL2B treatment disrupts integrin β4/pSrc/p53 axis in xenograft tumors. Two independent experiments were carried out with similar results.** j** The exo-circRABL2B treatment caused cell death in two patients-derived LCOs. Viable and dead cells were stained by calcein AM (green) and EthD-1 (red), respectively. **k** Quantification of circRABL2B in exosomes from xenograft tumor tissues of circRABL2B-overexpressed or control cells. **l** Summary of exosomal circRABL2B expression level in detectable samples. **m** Agarose gel assay for validating the samples with detectable or undetectable circRABL2B in plasma exosomes. Data are presented as Mean ± S.E. from 3-6 biologically replicates in d, e, f, g, h, and k. *P* values are calculated by unpaired *t* test or Two-way ANOVA test.

Exosomes were efficient carriers to transfer cargoes, including circRNAs in intercellular communication and nano-scale drug-delivery^20^. To evaluate the therapeutic potential of circRABL2B, we tested whether exosomes carrying high level of circRABL2B could suppress tumor growth* in vitro*, *ex vivo* and* in vivo*. We found that exosomes from culture medium of circRABL2B-overexpressing cells (i.e., exo-circRABL2B) have an explicit increment in circRABL2B level in comparison with that from medium of control cells (i.e., exo-vector; **Fig. 6a**). We also found that circRABL2B did not affect exosomes production or section, because there were no differences in count between exo-circRABL2B and exo-vector (**Fig. S8a**). After determining that exo-circRABL2B or exo-vector could be internalized by the receipt cells* in vitro* and tumors *in vivo* (**Fig. S8b, c**), we used the exo-circRABL2B or exo-vector from cell culture medium to treat PC9 cells, tumorigenic nude mice and patients-derived LCOs (**Fig. 6b**). After treatment, exo-circRABL2B significantly suppressed proliferation, clone formation, migration and invasion *in vitro* (**Fig. 6c-f**) and reduced tumor size and weight* in vivo* (**Fig. 6g**). Conclusively, the xenograft tumors after exo-circRABL2B treatment displayed more circRABL2B than those after exo-vector treatment (**Fig. 6h**). Meanwhile, exo-circRABL2B treatment decreased expressions of integrin β4 and pSrc, while increased expressions of p53 and cleaved PARP (**Fig. 6i**). Using the LCOs from two lung cancer patients, the exo-circRABL2B treatment consistently induced more tumor cell death as shown by the IF with calcein AM for viable cells and EthD-1 for dead cells (**Fig. 6j**). These data suggested that exosome-packaged circRABL2B has a potential for treating lung cancer.

### Exosomal circRABL2B in serum has potential role on early diagnosis of lung cancer

We have previously reported the diagnostic application of circRNAs in exosomes on lung cancer^21^. Here, to determine the diagnostic role of exosomal circRABL2B on lung cancer, we first tested the expression of circRABL2B in tissue exosomes from preceding xenograft tumors in Fig 2m. The Ct value of circRABL2B was significantly lower in exosomes from circRABL2B-overexpressed xenograft tumors than that from controls (**Fig. 6k**), which means that circRABL2B-overexpressed xenograft tumors released more circRABL2B into exosomes. We therefore tested circRABL2B in plasma exosomes of 106 early-stage (i.e., stage I and II) lung cancer patients and 94 healthy controls. The detectable rate of exosomal circRABL2B in plasma was extremely lower in the patients than in the controls (15.1% vs. 52.1%, *P* < 0.001). The detection rate is higher than the exoRBase database record (http://www.exorbase.org/exoRBaseV2/browse/toIndex?kind=circRNA).With regard to the detectable samples, the expression of exosomal circRABL2B was also lower in patients than in controls (**Fig. 6i**). Results from the Agarose gel assay successfully validated the quantitative real-time PCR (qPCR) results (**Fig. 6m**). We regarded undetectable circRABL2B as positive and detectable circRABL2B as negative, and found that serum exosomal circRABL2B exerted a sensitivity as 0.849 (95%CI = 0.763-0.909). These data suggest that circRABL2B in plasma exosomes has a potential application in the early diagnosis of lung cancer.

### EIF4a3 involves the biogenesis of circRABL2B in lung cancer cells

**Figure 7:**
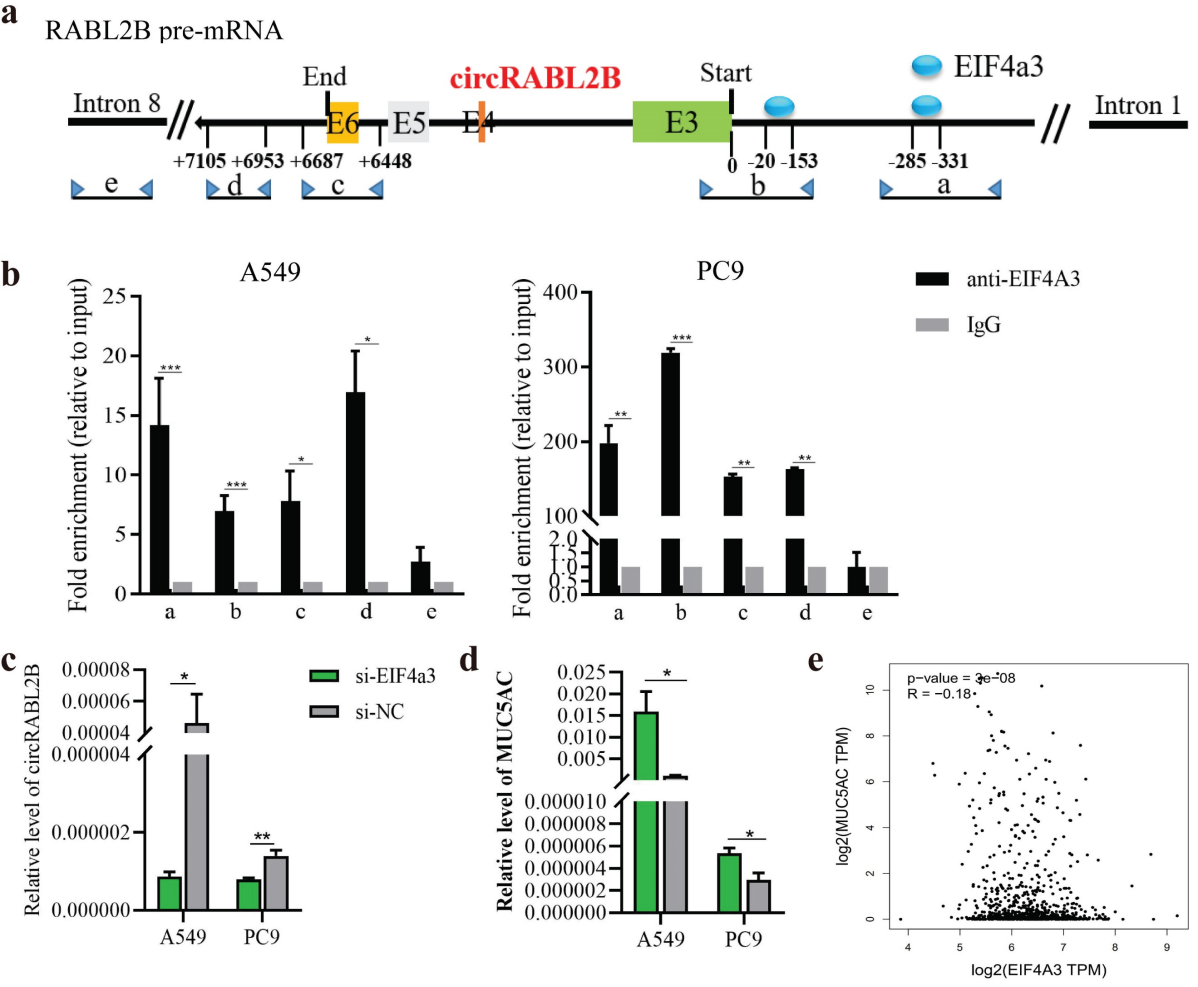
** EIF4a3 regulates circRABL2B expression. a** Schematic diagram showing the predicted binding sites of EIF4a3 in the upstream of circRABL2B pre-mRNA and the target sequences for CHIP assays.** b** CHIP assays for analyzing binding abilities of EIF4a3 on circRABL2B pre-mRNA in lung cancer cells. **c, d** Levels of circRABL2B (c) and MUC5AC (d) in lung cancer cells after EIF4a3 knockdown. **e** Analysis of TCGA data of lung cancer showing a correlation between EIF4a3 and MUC5AC expressions in lung cancer patients. Data are presented as Mean ± S.E. from 3-4 biologically replicates. *P* values are calculated by unpaired *t* test.

The biogenesis of circRNA is highly dependent on the transcription levels of circRNA-producing pre-mRNA and post- or co-transcriptional regulation^22^. Both RABL2B and circRABL2B are derived from RABL2B pre-mRNA, and RABL2B mRNA was also significantly downregulated in the lung cancer tissues in comparison with the normal tissues, being similar to the TCGA data (**Fig. S9a, b**). There was no significant effect of RABL2B on lung cancer survival and MUC5AC by analyzing the TCGA data (**Fig. S9c, d**). Meanwhile, knock-down of RABL2B did not affect proliferation, cloning formation, migration and invasion of lung cancer cells (**Fig. S9e-h**). These data suggests that the mother gene of circRABL2B, RABL2B may be not implicated in lung cancer development. By inquiring about the circinteractome (https://circinteractome.nia.nih.gov/index.html), we discovered several binding sites for EIF4a3 at the flanking regions of RABL2B pre-mRNA. EIF4a3 is an exon-junction complex helicase that participates in the formation of several circRNAs^23^, ^24^. We defined two putative EIF4a3 binding sites in the upstream of circRABL2B pre-mRNA as a and b, the sequence stretching over the end of circRABL2B as c, the sequence at the downstream of circRABL2B as d, and the sequence on RABL2B pre-mRNA intron 8 as e (**Fig. 7a**). The CHIP assay not only confirmed that EIF4a3 is indeed bound to the two putative sites but also occupied the sites stretching over the end of circRABL2B and the downstream of circRABL2B (**Fig. 7b**). Furthermore, siRNAs-mediated knockdown of EIF4a3 (**Fig. S10**) significantly decreased circRABL2B expression and increased MUC5AC level in lung cancer cells (**Fig. 7c, d**). Consistently, TCGA data demonstrated a negative correlation between expressions of EIF4a3 and MUC5AC in lung cancer (**Fig. 7e**). These data suggested that circRABL2B is downregulated at the transcription level and EIF4a3 helps the formation of circRABL2B.

## Discussion

Therapeutic targets-related circRNAs are promising biomarkers for individual treatment selection and subsidiary anti-cancer drugs or drug targets^25^. MUCs have been recognized as hot targets for anti-cancer immunotherapies for years, and some anti-MUC drugs have shown clinical prospects in treating advanced cancer^26^, ^27^. Nevertheless, there was no revelation as to lung cancer, whether and which circRNAs affect MUCs function. In the current study, we report that circRABL2B is negatively correlated with MUC5AC and downregulated in lung cancer, which indicates poor survival. CircRABL2B physically interacts with YBX1 to inhibit MUC5AC expression and impoverishes lung cancer cell stemness via affecting MUC5AC-induced CD44/pSrc/p53 pathway.

Being well-established as a cancer-promoting molecule, MUC5AC has overexpressed in several cancers, which indicates a poor prognosis of lung adenocarcinoma patients^28^-^30^. Being consistent, here we confirmed that MUC5AC acts as a tumor promoter. MUC5AC is overexpressed in lung cancer tissues and associated with poor survival of lung cancer patients. Knock-down of MUC5AC also inhibited lung cancer growth and migration. We also found one negative regulator of MUC5AC, that is circRABL2B, was also correlated to the patients' prognosis. The patients who expressed both low circRABL2B and high MUC5AC displayed the worst survival rate, suggesting circRABL2B and MUC5AC to be bi-target for treatment of lung cancer. Knock-down of MUC5AC restored the cell malignant phenotypes caused by circRABL2B inhibition, indicating that MUC5AC was a major mediator of circRABL2B's anti-cancer function.

Most studies showed that circRNAs act as a ceRNA in lung cancer^31^, ^32^. Here, we demonstrated that circRABL2B exerts function underlying the mechanism not through ceRNA but via interaction with YBX1. YBX1 functions as both DNA binding protein (DBP) and RBP and has been implicated in numerous cellular processes^33^. YBX1 was marked as an oncogene as regrading to nuclear localization and transcription factor in several cancers^34^, ^35^. However, our immunofluorescent localization showed that YBX1 enriched in the cytoplasm in lung cancer cells. YBX1 physically interacted with circRABL2B to decrease MUC5AC expression and affected integrin β4/pSrc/p53 axis, while knock-down of YBX1 counteracted the inhibitory effect of circRABL2B on lung cancer cells. The negative correlation between YBX1 and MUC5AC in lung tumors of TCGA further strengthens our finding. Acting as a RBP, previous studies also showed that cytoplasmic YBX1 interacted with other circRNAs such as circFOXK2 and circFAT1 to promote or inhibit cancer progression^36^, ^37^, suggesting that cytoplasmic YBX1 provides a scaffold for circRNAs to exert their function. With this background, researchers need to be alert to negative side effects when exploiting drugs targeting on anti-nuclear YBX1 since YBX1 was recognized as a drug target for many years^38^.

The GSEA on the gene chip data suggested regulation of circRABL2B on p53 signaling. Indeed, previous researches have indicated MUC5AC promotes cancer progression by enriching self-renewing cancer stem cells via integrin β4/pSrc/p53 axis^19^, ^39^. Instead, here we found circRABL2B, which decreases MUC5AC expression, acts to decrease the proportion of SP cells in lung cancer. We further confirmed that circRABL2B inhibits integrin β4/pSrc pathway and enhances p53 expression. Therefore, our data discovered a novel circRNA participating in cancer cell stemness. We also found that circRABL2B promotes the erlotinib sensitivity. Erlotinib is a type of targeted cancer drug that targets mutated epidermal growth factor receptor (EGFR). Previously, a phase IIA Trial Testing showed that erlotinib may diminish MUC5AC expression in tumor tissues^40^. Also, MUC5AC is one of the main downstream factors that can be activated by the EGFR signaling pathway^41^. Here, the *in silico* analysis demonstrated that MUC5AC promotes erlotinib resistance. Thus, being an upstream regulator of MUC5AC, it is of considerable value to target circRABL2B to conquer erlotinib resistance.

In recent years, the use of exosomes as both biomarkers and biological delivery vehicles has been of considerable interest for cancer diagnosis, prognosis evaluation and treatment. According to the exoBase 2.0 (http://www.exorbase.org/; last accessed: Sep 23, 2021), a total of 84,044 circRNAs were documented in exosomes, including circRABL2B. However, little is known about the biological value of exosomal circRABL2B. Here, we first expose that exosomal circRABL2B can be released by lung tumor cells* in vitro* and* in vivo* and phagocytosed by receipt cells, causing a clear reduction in proliferation, colony formation, migration and invasion of receipt cells. More importantly, exosomes carrying a high level of circRABL2B suppress tumor growth* in vivo* and* ex vivo*. Furthermore, we show that serum exosomes from early-stage lung cancer patients display a remarkably low detection rate of circRABL2B compared to healthy controls, and exosomal circRABL2B level in serum exhibits a considerable sensitivity with regard to distinguishing the patients from the healthy controls. This evidence supports a considerable exploration of exosomal circRABL2B on treating lung cancer and early diagnosis.

## Conclusion

CircRABL2B is a previously undiscovered natural antagonist for MUC5AC, which functions to suppress lung cancer progression via MUC5AC/integrin β4/pSrc/p53 axis and cell stemness impoverishment. Exosome-packaged circRABL2B has potential for use in anti-MUC treatment and early diagnosis of lung cancer. These data imply that circRABL2B may be an attractive target for the prevention and treatment of lung cancer.

## Supplementary Material

Supplementary methods, figures and tables.

## Figures and Tables

**Table 1:** Associations of MUCs and related circRNAs with lung cancer survival.

Gene/circRNA	n (%)	Death	MST (months)	Log-rank *P* value	HR (95%CI)*^ a^*	COX mode *P* value *^a^*
MUC5AC						
Low	45(31.9)	35	18	0.039	1.00(ref.)	
High	96(68.1)	80	12		1.46(0.97-2.21)	0.068
MUC5B						
Low	70(49.6)	54	14	0.467	1.00(ref.)	
High	71(50.4)	61	13		1.07(0.73-1.58)	0.731
circRABL2B						
High	96(68.1)	75	16	0.016	1.00(ref.)	
Low	45(31.9)	40	12		1.38(0.91-2.08)	0.129
CircSULF2						
Low	70(49.6)	58	13	0.702	1.00(ref.)	
High	71(50.4)	57	14		1.10(0.74-1.64)	0.632
MUC5AC+ circRABL2B						
Low + High	33(23.4)	24	21	0.023	1.00(ref.)	
High + High	63(44.7)	51	12		1.55(0.94-2.56)	0.087
Low + Low	12(8.5)	11	14		1.57(0.74-3.34)	0.236
High + Low	33(23.4)	29	12		2.00(1.12-3.57)	0.020

Abbreviations: MST, median survival time; HR, hazard ratio; *^a^* Cox regression analysis was adjusted for age, sex, smoking status, family history of lung cancer and clinical stage.

## Abbreviations

MT: Molecular therapy
ICB: immune checkpoint blockade therapy
MUC: Mucin
CircRNA: Circular RNA
KEGG: Kyoto encyclopedia of genes
GSEA: gene set enrichment analysis
SP: Side population
LCO: lung cancer organoid
IF: immunofluorescence
FISH: fluorescence in situ hybridization
RIP: RNA immunoprecipitation
ChIP: chromatin immunoprecipitation
