# Supplementary material for: EIF4a3-regulated circRABL2B regulates cell stemness and drug sensitivity of lung cancer via YBX1-dependent downregulation of MUC5AC expression
Source: Int J Biol Sci. 2023 May 21;19(9):2725–39. doi: 10.7150/ijbs.78588 (PMC10266078; doi:10.7150/ijbs.78588)
Supplement: Supplementary file 1 — Supplementary methods, figures and tables. [file ijbsv19p2725s1.pdf]

Supplementary Materials

Supplementary Methods

Cell culture

All human lung cancer cell lines and non-tumorigenic lung epithelial cell lines were purchased from the Cell Bank of Type Culture Collection of the Chinese Academy of Science (Shanghai Institute of Cell Biology, Shanghai, China). All cells were cultured in RPMI1640 medium (GIBCO, Thermo Fisher Scientific, Waltham, MA) supplemented with 10% fetal bovine serum (FBS, GIBCO).

Gene expression test

Expression levels of indicated targets were tested by routine quantitative real-time PCR (qPCR) and western blotting (WB). The primers as well as probes for each target used in this study are listed in **Table S2**, except for MUC5AC, MUC5B, and MUC3A, which were directly purchased from Thermo Fisher Scientific. The information about antibodies used is listed in **Table S3**. Moreover, the MUC5AC level was also quantitated in cell culture supernates using the human MUC5AC enzyme-linked immunosorbent assay (ELISA) Kit (CUSABIO, China).

Ribonuclease R treatment

Total RNA of A549 cells was treated with or without Ribonuclease R (3-4 U/μg RNA) for 30 min at 37°C. The products were then submitted to PCR and agarose gel electrophoresis.

Vector construction and cell transfection

For circRNA overexpression, the linear sequence of circRABL2B flanked by the cyclization sequence was amplified and inserted into the pLCDH-ciR plasmid. For circRNA

knockdown, short hairpin RNAs (shRNAs) targeting circRABL2B and circSULF2 were synthesized and inserted into the pLshRNA plasmid. For gene knockdown, siRNAs targeting MUC5AC, YBX1, RABL2B and EIF4a3 were purchased from Ribo^{TM} Company (Guangzhou, China). To generate stable overexpression cells and knockdown cells, the transfection of plasmids was performed using Lenti-Pac HIV Expression Packaging Kit (Genecopoeia, Rockville, MD), and the harvestable virus particles were used to infect A549 and PC9 cells. To knockdown MUC5AC, YBX1, and EIF4a3, the transfection of mixed siRNAs was performed using the Lipofectamine 2000 Kit (Invitrogen).

In vitro cell behavior assays

To test cell proliferation, approximately 1×10^3 cells were seeded in 100 μl culture media in 96-well plates and cell viability was analyzed using Cell Counting Kit-8 (Beyotime, Shanghai, China). To test cell oncogenicity, approximately 200 cells were seeded in 2 ml culture media in 6-well plates for 7-14 days. Then the cells were stained using Crystal Violet and counted. To test cell migration and invasion, approximately 2×10^5 cells were seeded in Corning Transwell insert chambers (Corning, New York) and BD BioCoat Matrigel invasion Chambers (BD Biosciences, Bergen, NJ), respectively. Cells migrated through the membrane were fixed, stained, and counted under a light microscope. To test the cell cycle, approximately 2×10^4 cells were incubated with 10 ul propidium iodide (PI) and submitted to flow cytometry (FCM) on FACScan (BD Biosciences). To test the cell apoptosis, approximately 1×10^5 cells were stained with 5 μl FITC-Annexin V and 10 μl 7AAD and then submitted to flow cytometry (FCM).

In vivo tumorigenesis assay

To test xenograft *in vivo*, 6–8-week-old female BALB/c nude mice were used. Approximately 5×10^6 circRABL2B overexpressed A549 cells or control A549 cells, or circRABL2B knockdown PC9 cells or control PC9 cells were injected subcutaneously into the dorsal flank of mice. After 2-to-3 weeks, all mice were sacrificed and tumor volume was measured using a caliper as $1/2\times\text{length}^2\times\text{width}$. Tumors were then surgically extracted and weighted.All experiments and procedures involving animals were conducted in accordance with guidelines approved by the Laboratory Animal Center of Guangzhou Medical University.

Immunofluorescence (IF) staining and RNA fluorescence in situ hybridization (FISH)

To detect MUC5AC *in vitro*, A549 or PC9 cells were fixed, permeabilized, blocked and incubated at 4°C overnight with MUC5AC antibody. Then fixed cells were rinsed in Dulbecco's Phosphate Buffered Saline (DPBS; Thermo Fisher Scientific), which was followed by a reaction with AlexaFluor 594-conjugated Goat Anti-Rabbit IgG (Abcam) for 1 h and DAPI (Invitrogen) for 10 min.

To determine co-localization of circRABL2B with YBX1 or SFPQ, A549 or PC9 cells were fixed, permeabilized, blocked and incubated at 4°C overnight with YBX1 or SFPQ antibody. Subsequently, fixed cells were rinsed in DPBS and incubated at room temperature with AlexaFluor 488-conjugated Goat Anti-Rabbit IgG. Then cells were washed with DPBS three times, which was followed by RNA-FISH using Ribo™ FISH Kit (Guangzhou, China) according to the operating manual. Cells were fixed, permeabilized, and prehybridized for 30 min. Then hybridization was carried out with circRABL2B probes that were designed and synthesized by Ribo™ Company at 37°C in the dark overnight. After being washed in wash buffer at 42°C and DPBS, cells were incubated with DAPI for 10 min. Imaging was

performed on an LSM980 confocal laser scanning biological microscope (ZEISS, Jena, Germany). Antibody information is listed in **Table S3**.

RNA pull-down, RNA immunoprecipitation (RIP), and chromatin immunoprecipitation (ChIP)

RNA pull-down assay was performed using BersinBio^{TM} RNA pull-down Kit (BersinBio, Guangzhou, China) according to the manufacturer's instructions. Briefly, 2×10^7 cells were sonicated in 1.7 ml RIP buffer, supplemented with a 17 μl protease inhibitor. After centrifugation, the cell supernatant was incubated with agarose beads and then incubated with Streptavidin magnetic beads-treated biotinylated DNA oligo probes against circRABL2B or LacZ control, supplemented with auxiliary reagents for 2 h at room temperature. The beads were washed with ice-cold NT2 buffer five times and eluted with Protein elution buffer. The retrieved proteins were used for mass spectrometry or WB analysis. Mass spectrometry was finished by a commercial corporation (BersinBio). Sense, antisense of full length circRABL2B and truncated circRABL2B with deletion of predicted YBX1-binding sequences (nucleotides 118-134), and sense, antisense of 5'-end MUC5AC transcript (nucleotides 1-1950), and truncated 5'-end MUC5AC transcript with deletion of predicted YBX1-binding sequences (nucleotides 225-784), were cloned into a pMD18-T vectors (BersinBio) and were biotinylated with a Pierce RNA 3'-End Desthiobiotinylation Kit (Thermo Fisher Scientific, Waltham, MA).

RIP was performed using BersinBio^{TM} RNA Immunoprecipitation Kit (BersinBio) according to the manufacturer's instructions. Briefly, cells were lysed using RNA lysis buffer and then incubated with the RIP washing buffer containing the magnetic beads conjugated

with antibodies of indicated proteins or negative control IgG at 4°C, overnight. The beads were washed six times. The immunoprecipitated RNAs were extracted using phenol-chloroform-isoamylol and were used for RT-qPCR.

ChIP was performed using a high-sensitivity ChIP kit (Abcam). Briefly, tissue cultures were fixed, quenched and lysed. Then the chromatin DNA was sheared to fragments. The samples were incubated with a 0.8 μg primary antibody against EIF4a3, or non-immune IgG and mixed with ChIP reaction buffer overnight at 4°C. After washing, DNA fragments were eluted and used for qRT-PCR. Fold enrichment was calculated as a ratio of the ChIP sample amplification efficiency to non-immune IgG, as suggested by the manufacturer. Primer information is listed in **Table S2**.

Exosome isolation, identification

Tissue exosomes were isolated from the xenografts of nude mice following standard centrifugation steps. The samples were treated by ExoQuick-TC (System Biosciences, Palo Alto, CA) at 4°C and then were centrifuged at 1500 g for 30 min. The pellet was re-suspended in PBS and further centrifuged at 20,000 g for 2 h. The pellet was diluted in PBS. Transmission electron microscopy was performed to identify exosomes.

Table S1. Frequency distributions of demographics in studied patients.

Variables		Number of cases (%)
Age (years)	< 60	86 (61.0%)
	≥ 60	55 (39.0%)
Sex	Male	93 (66.0)
	Female	48 (34.0)
Smoking status	Ever	85 (60.3)
	Never	56 (39.7)
Family history of cancer	Yes	17 (12.1%)
	No	124 (87.9%)
Stages	I + II	44 (31.2%)
	III + IV	97 (68.8%)
Histological types	Adenocarcinoma	65 (46.1%)
	Squamous cell carcinoma	45 (31.9%)
	Other [a]	31 (22.0%)

[a] Mixed-cell, magnocellular or undifferentiated carcinoma of lung.

Table S2. Sequence information of primers and probes used in this study.

Targets	Primers sequences (5'-3')	Probes
Taqman qPCR		
CircRABL2B	CCACGCCTGCATCATGAC (Forward)	FAM-CAATACCCTCCCC TCCCTTGGGC-BHQ
	TCTGCCATTGGCTCCTAGCT (Reverse)	
GAPDH	ACAGCCTCAAGATCATCAGCA (Forward)	FAM-TCCTGCACCACCA ACTGCTTAGC-BHQ
	ATGAGTCCTTCCACGATACCA (Reverse)	
PCR or SYBR qRT-PCR		
CircRABL2B	CACGCCTGCATCATGACAGT (Forward)	
	CCAGGCAGATGATCTTCACG (Reverse)	
CircSULF2	CTTTGTGGGTACGGGCCAAG (Forward)	
	CTAGCGTGAATCAGAACCTCGC (Reverse)	
RABL2B	CTGGTCTCTCCAGCCCTCA (Forward)	
	TTGCCATCTTTCCGTGCTTC (Reverse)	
YBX1	GAAGTGATGGAGGGTGCTGACAAC (Forward)	
	TCTAGGCTGTCTTTGGCGAGGAG (Reverse)	
U6	CTCGCTTCGGCAGCACA (Forward)	
	AACGCTTCACGAATTTGCGT (Reverse)	
EIF4a3	ATGGCGACCACGGCCACGATG (Forward)	
	TCCCGCAGGCCCATGGTGTCG (Reverse)	
β-actin	CATGTACGTTGCTATCCAGGC (Forward)	
	CTCCTTAATGTCACGCACGAT (Reverse)	
EIF4a3 binding sites		
a	TCATGGTGAATGCCCAGTAA (Forward)	
	ATGCTGCTCCTCACGTTACC (Reverse)	
b	GGGTGCGCAGGACTTAACTA (Forward)	
	GGAGGGGAGGGTATTGTCAC (Reverse)	
c	ACTTGCACAGAAGTGCACCA (Forward)	
	CTCCCACAACACCCAGACTT (Reverse)	
d	CGCTGGGACTGAGTAGATGC (Forward)	
	TTCTCTTGGAGCGACTGCTT (Reverse)	
e	CTGCAGGTGTGATGGAGAGA (Forward)	
	GCAACCTTACCCCTCCATTT (Reverse)	

Table S3. List of antibodies used in this study.

Antibody	Catalog No. (Company)	Concentration	Application
Rabbit anti-MUC5AC	[EPR16904] (ab198294) (Abcam)	1:250	IF
Rabbit anti-YBX1	[EP2708Y] (ab76149)(Abcam)	1:100	IF
Rabbit anti-SFPQ	(ab99357) (Abcam)	1:100	IF
Rabbit anti-YBX1	[EP2708Y] (ab76149)(Abcam)	1:3000	WB
Rabbit anti-SFPQ	(ab99357) (Abcam)	1:3000	WB
Mouse anti-CD44	#3570 (Cell Signaling Technology)	1:1000	WB
Rabbit anti-EpCAM	(ab71916) (Abcam)	1:1000	WB
Mouse anti-p53	(DO-1): sc-126(SANTA CRUZ BIOTECHNOLOGY, INC)	1:200	WB
Mouse anti-Integrin β4	[M126] (ab29042) (Abcam)	1:1000	WB
Mouse anti-pSrc	((Tyr416) Antibody #2101) (Cell Signaling Technology)	1:1000	WB
Rabbit anti-Src	#2108(Cell Signaling Technology)	1:1000	WB
Rabbit anti-Cleaved PARP	(Asp214) XP® Rabbit mAb #9544(Cell Signaling Technology)	1:1000	WB
Rabbit anti-GAPDH	[EPR16891] (Abcam)	1:10000	WB

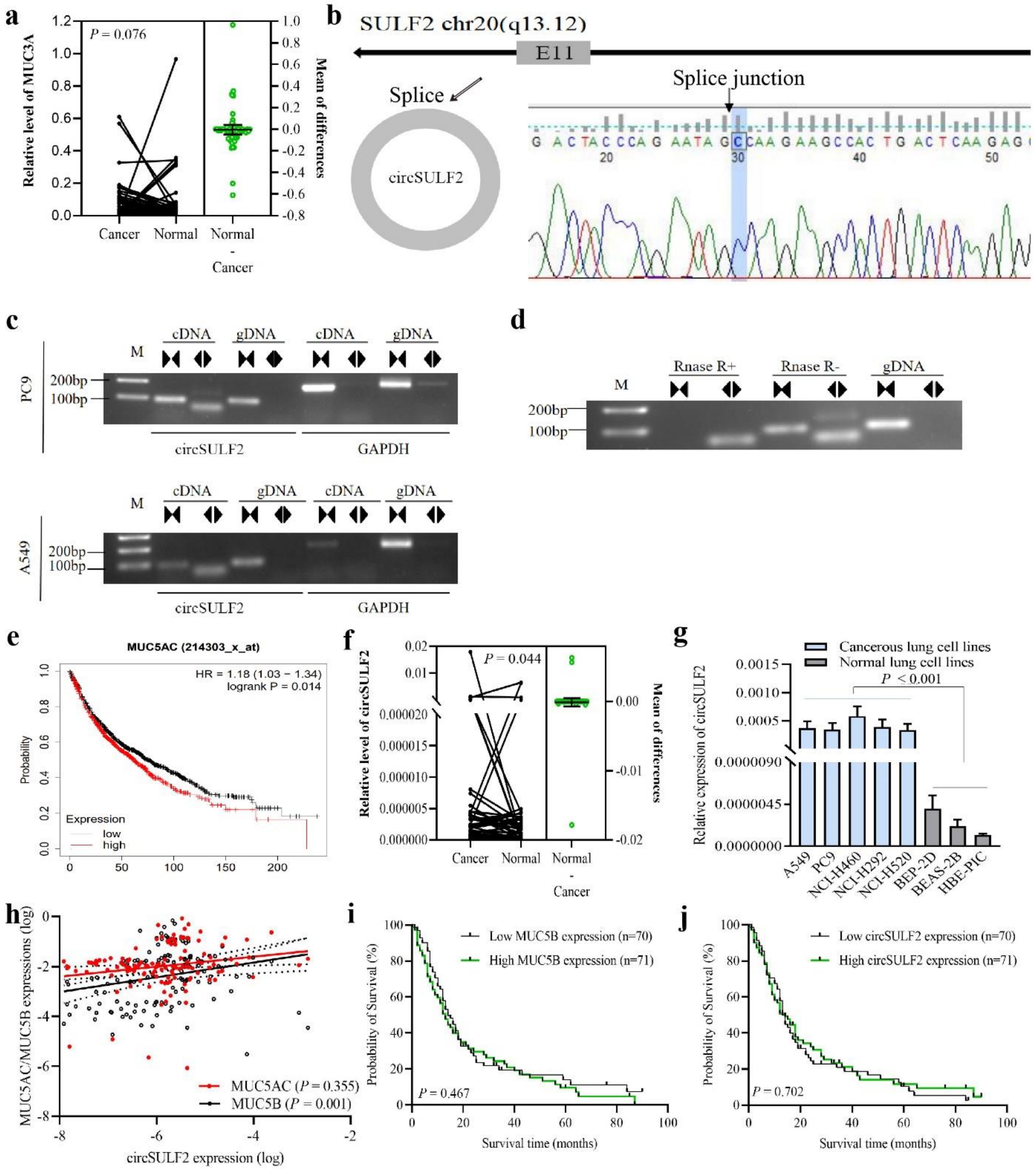

Fig. S1: circSULF2 is upregulated in lung cancer but not associated with lung cancer survival. a Analysis for mRNA levels of MUC3A in additional 68 paired samples of lung cancer. **b-d** Schematic representation and characterization of circSULF2 formation. The backsplice junction site of circSULF2 was identified by Sanger sequencing (b). PCR amplification of circSULF2 and GAPDH with cDNA or

gDNA in A549 and PC9 (c). PCR analysis for circSULF2 after treatment with or without RNase (d). **e** The Kaplan-Meier survival curve for lung cancer patients according to expressions of MUC5AC in KM plotter database. **f** Analysis for circSULF2 levels in 68 paired samples of lung cancer. The differences between cancer and normal are presented as Mean ± S.E. and *P* values are calculated by Wilcoxon matched-pairs signed rank test. **g** Determination of circSULF2 expression in lung cell lines. n = 6-7 biologically independent samples for each cell line. Data are presented as Mean ± S.E. The *P* value is tested by unpaired *t* test. **h** Correlations between expressions of MUC5AC, MUC5B and circSULF2. *P* values are calculated by Spearman correlation test. **i, j** Kaplan-Meier survival curves for lung cancer patients according to expressions of MUC5B (i) and circSULF2 (j). *P* values are calculated by Log-rank test.

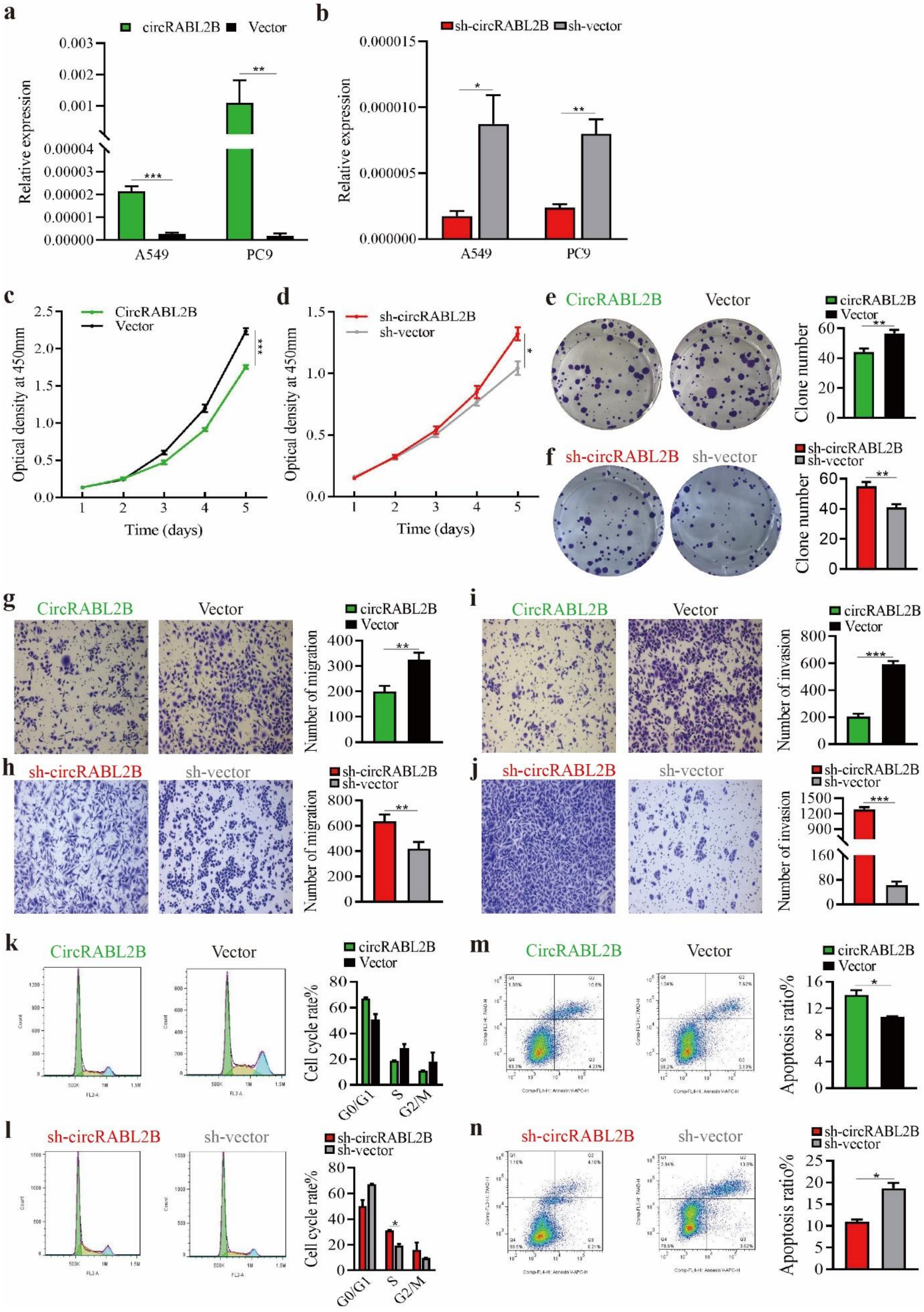

Fig. S2: CircRABL2B inhibits PC9 malignant phenotypes. a Analysis for

circRABL2B expression in lung cancer cells with circRABL2B overexpression (circRABL2B) versus vector control (vector). **b** Analysis for circRABL2B expression in lung cancer cells with circRABL2B knock-down (sh-circRABL2B) versus shRNA vector control (sh-vector). **c-n** Assays for testing cell proliferation (CCK8; c, d), colony formation (soft agar; e, f), migration (Transwell; g, h) and invasion (Transwell; i, j), for measuring cell cycle (FCM; k, l), and for cell apoptosis (FCM; m, n) in PC9 cells with circRABL2B overexpression (circRABL2B) versus vector control (vector), or cells with circRABL2B knock-down (sh-circRABL2B) versus shRNA vector control (sh-vector). Data are presented as Mean ± S.E from 3-6 biological replicates for different assays. *P* values are calculated by Two-way ANOVA test in c, d and unpaired *t* test in e-n. * $P < 0.05$, ** $P < 0.01$, *** $P < 0.001$.

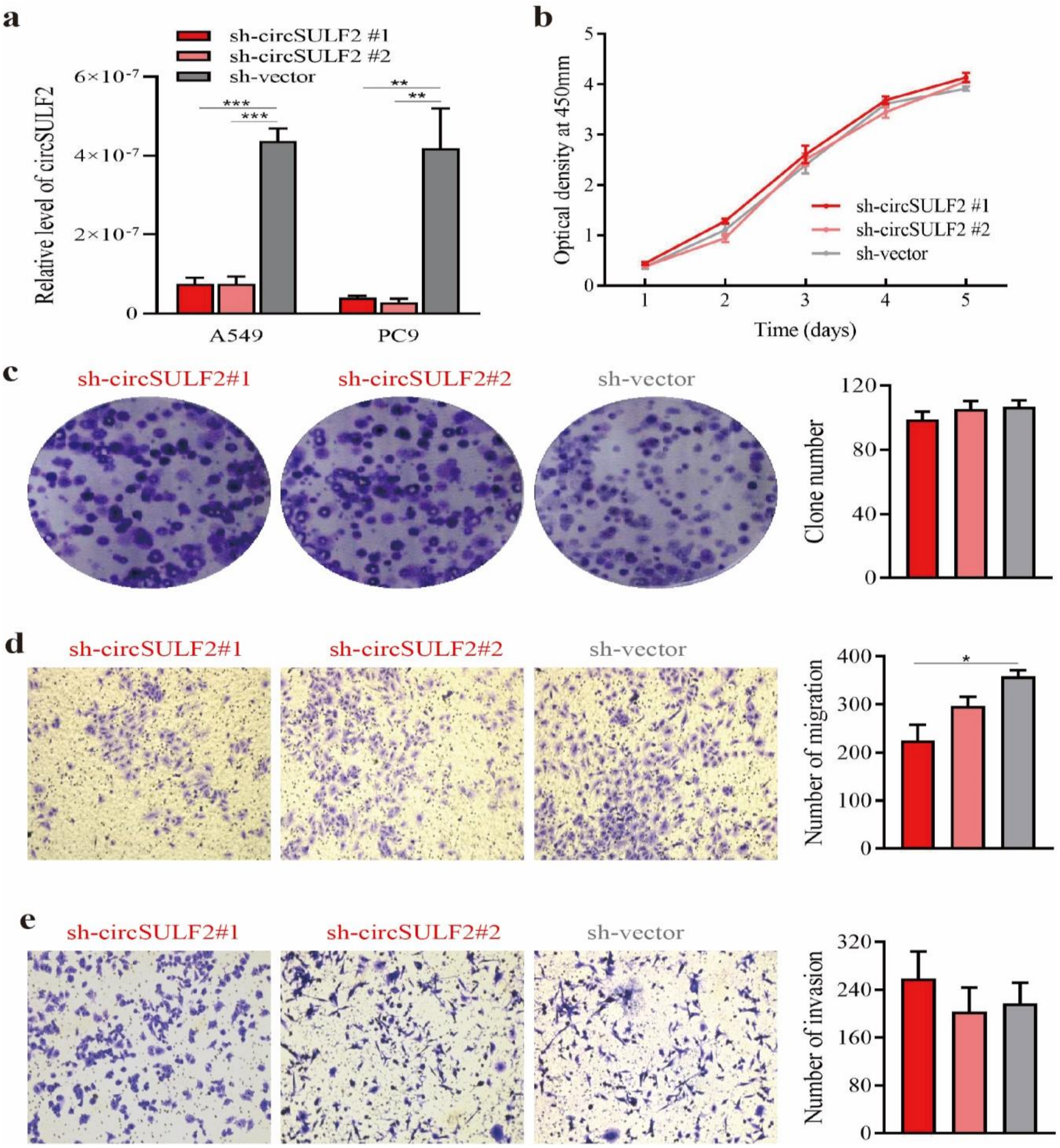

Fig. S3: CircSULF2 fails to affect malignant phenotypes of lung cancer cells. a Analysis for circSULF2 expression in lung cancer cells after transfection with shRNA vectors. **b-e** Assays for testing cell proliferation (b), colony formation (c), migration (d) and invasion (e) in A549 cells with circSULF2 knockdown (sh-circSULF2) versus shRNA vector control (sh-vector). Data are presented as Mean ± S.E. from 3-6 biologically replicates for different assays. *P* values are calculated by Two-way ANOVA test in b or unpaired *t* test in c-e. * $P < 0.05$.

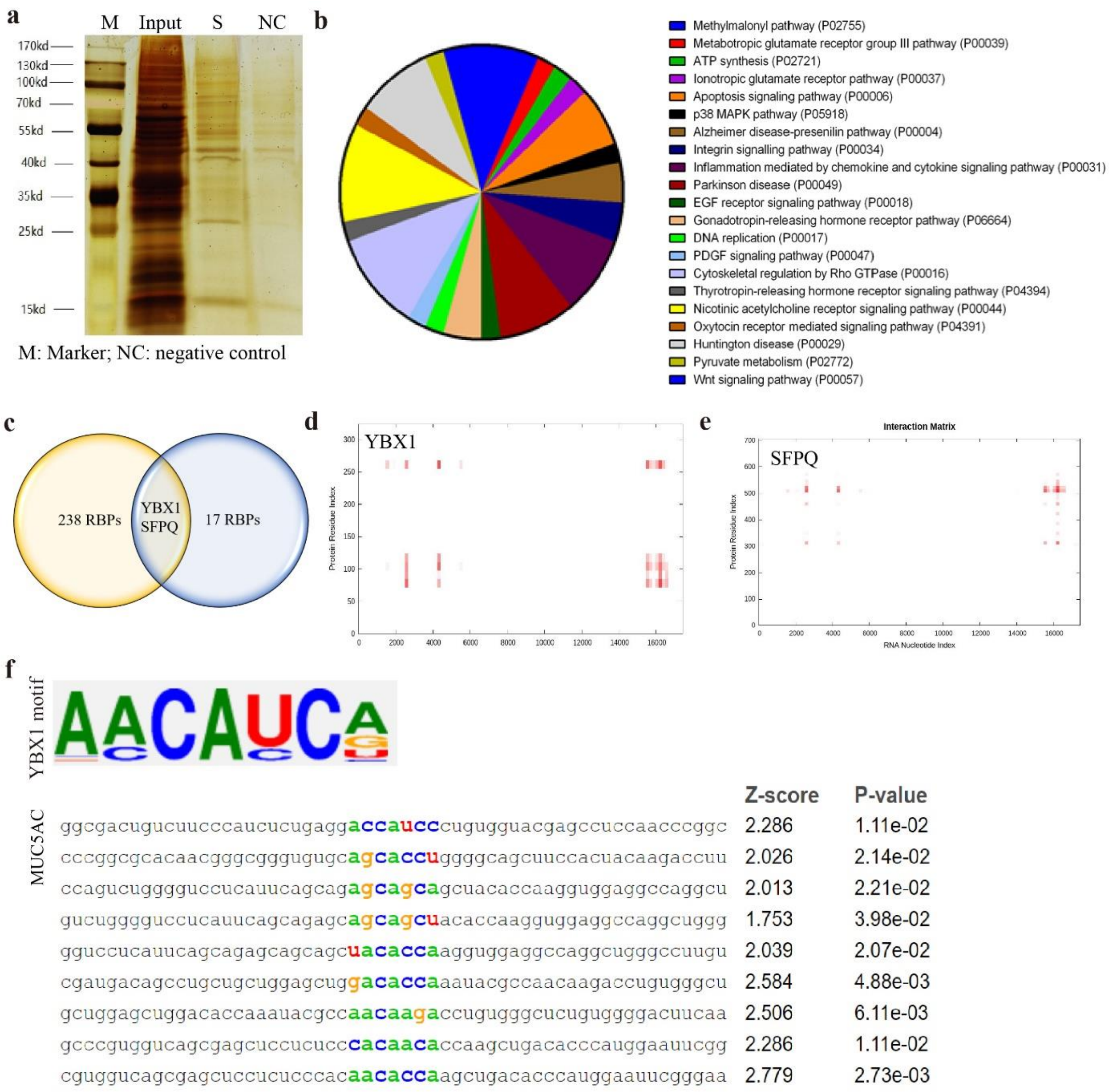

Fig. S4: RBPs associated with circRABL2B and MUC5AC. a Silver staining SDS-PAGE gel for RBPs fractions. **b** KEGG analysis for RBPs. **c** Numbers of RBPs associated with circRABL2B and MUC5AC. **d, e** Interaction between YBX1 (d) or SFPQ (e) and MUC5AC mRNA. **f** *In silico* analysis for binding site visualization at 5’-end of MUC5AC for YBX1. RBP: RNA binding protein.

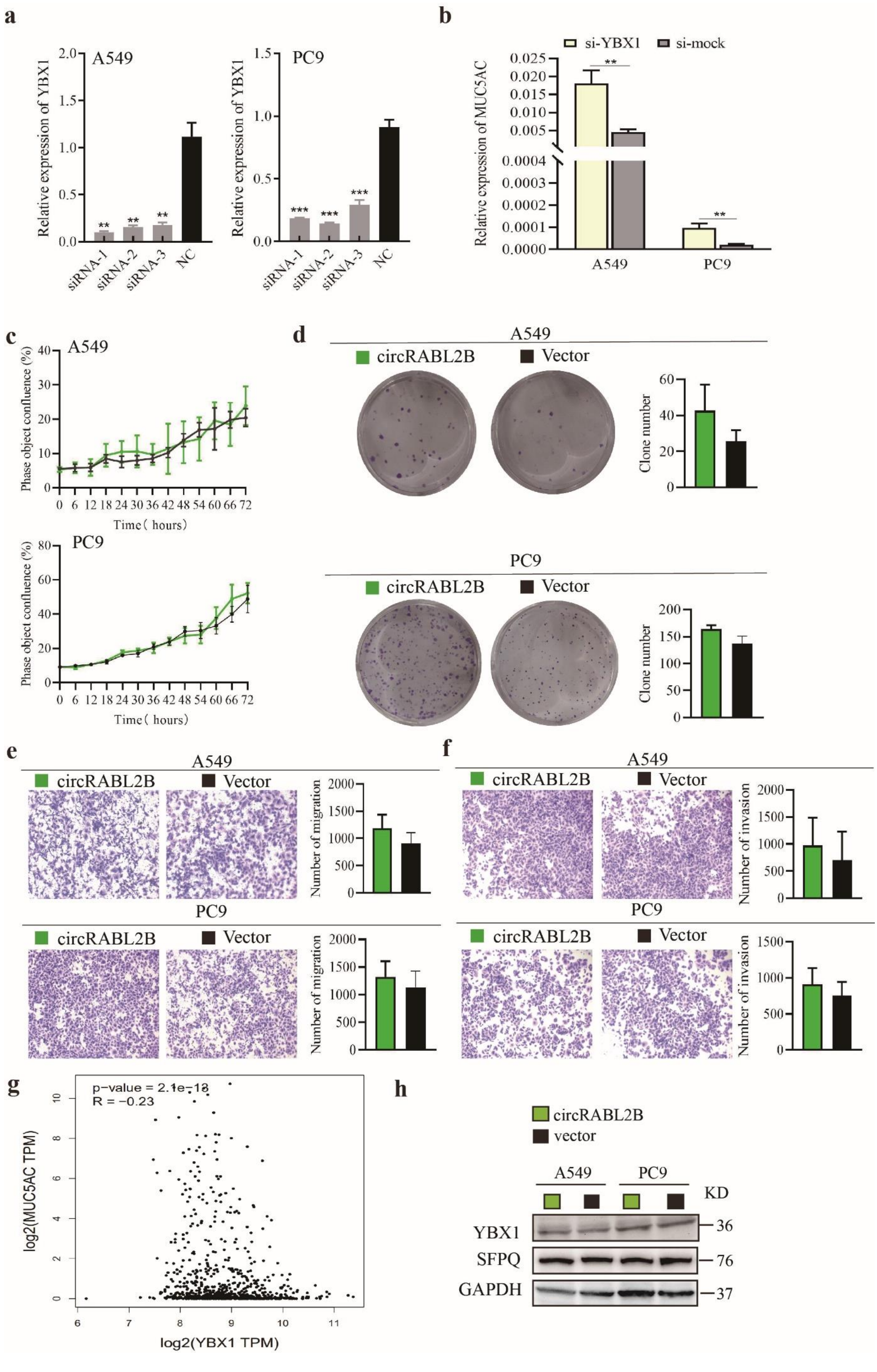

a
A549
PC9
Relative expression of YBX1
siRNA-1
siRNA-2
siRNA-3
NC
b
si-YBX1
si-mock
Relative expression of MUC5AC
A549
PC9
c
Phase object confluence (%)
Time(hours)
d
circRABL2B
Vector
Clone number
e
Number of migration
f
Number of invasion
g
p-value = 2.1e-18
R = -0.23
log2(MUC5AC TPM)
log2(YBX1 TPM)
h
circRABL2B
vector
KD
YBX1
SFPQ
GAPDH

Fig. S5: YBX1 is essential in antitumor effect of circRABL2B but not a target of circRABL2B. **a** Knock-down of YBX1 by siRNAs. **b** Determination of MUC5AC expression after YBX1 knock-down. **c-f** Assays for testing cell proliferation (c), colony formation (d), migration (e) and invasion (f) in lung cancer cells with circRABL2B overexpression (circRABL2B) versus vector control (vector) in response to YBX1 knock-down. **g** Analysis of TCGA data showing a negative correlation between YBX1 and MUC5AC. **h** WB showing no change in protein expressions of YBX1 and SFPQ after circRABL2B overexpression. Data are presented as Mean ± S.E of experimental triplicates. P values are calculated by Two-way ANOVA test in c, or unpaired t test in a, b, d, e, f, or Spearman correlation test in g. * $P < 0.05$, ** $P < 0.01$, *** $P < 0.001$.

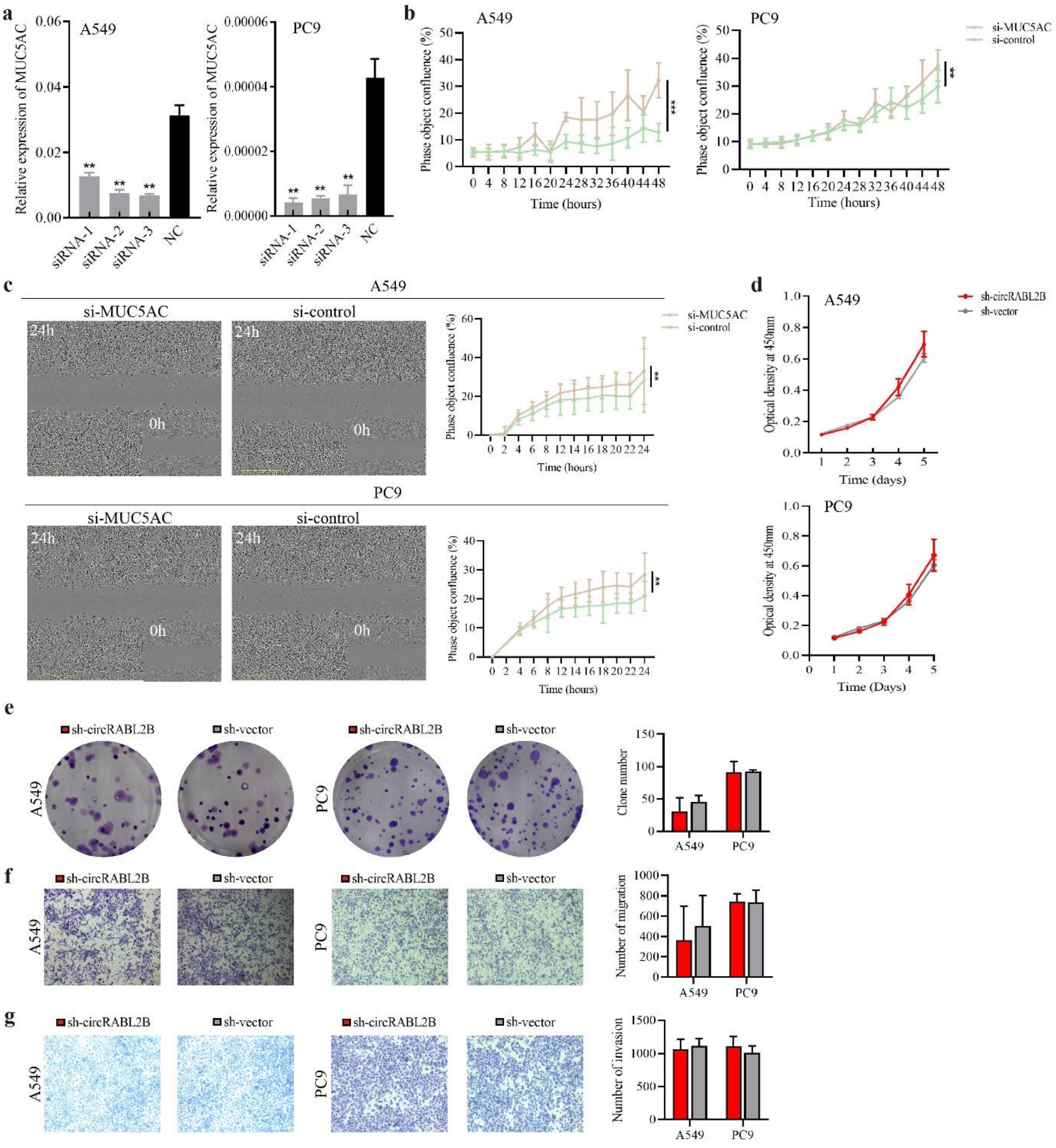

Fig. S6: Knockdown of MUC5AC rescues the inhibitory effect of circRABL2B on lung cancer cells. a Knock-down of MUC5AC by siRNAs. **b-c** Assays for testing cell proliferation (b) and migration (c) using the IncuCyte imaging system in response to MUC5AC knock-down. d-g Assays for testing cell proliferation (d), colony formation (e), migration (f) and invasion (g) in lung cancer cells with circRABL2B knock-down (sh-circRABL2B) versus vector control (sh-vector) in response to

MUC5AC knock-down. Data are presented as Mean ± S.E. from 3-6 biologically replicates for different assays. *P* values are calculated by unpaired *t* test in a, e-g and Two-way ANOVA test in b-d. ** $P < 0.01$.

a

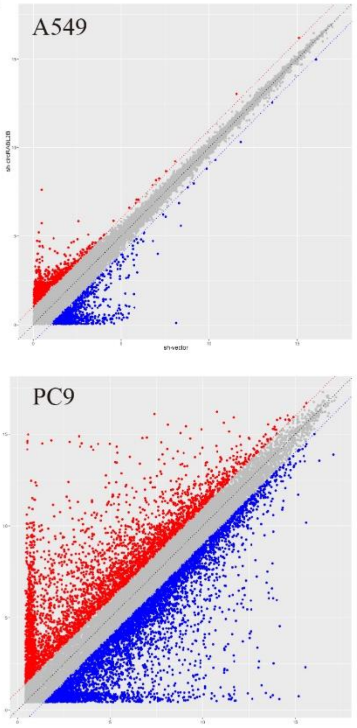

b

Genes	Log2 (Fold Change) A549	Log2 (Fold Change) PC9	Genes	Log2 (Fold Change) A549	Log2 (Fold Change) PC9
DSCAM	2.285826	2.363057	PRR32	-3.77016	-2.15246
ACPP	-2.78171	-2.40328	MUC5AC	3.426096	2.531892
PHGR1	-3.00396	-2.99693	MOB3B	-2.4704	-2.52752
OR10W1	-2.39419	-2.89066	GCNT1	-2.15958	-2.29869
CAPN8	-2.44544	-3.60227	PBX2	2.623307	2.344607
SH3BGRL2	-4.47293	-2.16965	LCNL1	-3.98283	-3.63361
SIRPD	2.767143	2.30389	DRD5	-3.06182	-2.61385
SAMD15	-2.37026	-2.21989	PPP4R4	3.788534	2.251248
APOF	-4.15361	-3.80292	DEFB103B	-2.5775	-2.90677
GPR137C	2.701487	2.954412	LOXL3	-2.74041	-2.06027
PLEKHS1	4.05503	2.034606	MUC4	-2.88141	-3.6131
FBXW2	-5.93318	-3.45752	RND2	2.267751	2.258679
GUCY1A3	2.994129	2.033408	ADAMTSL1	3.189268	3.053081
TNNT3	-3.06004	-2.18337	PGC	2.719143	2.086236
GRM2	-2.35509	-2.63035	SELPLG	-2.63964	-2.1193
USP2	-3.29716	-2.56148	SYTL4	-2.37904	-2.69185
SDR16C5	4.55577	2.35557	KLK12	-2.99064	-2.58002
ARL11	-4.18713	-2.14837	SYT10	2.343475	3.284093
ABAT	3.139335	2.120106	C11orf52	-2.69957	-3.31683
SLC13A1	-2.32488	-2.14221	PKD1	3.078396	2.128795
CEACAM3	-3.23501	-2.04547	ENG	-2.5852	-2.09317
EVPLL	-5.06922	-2.29192	KRTAP5-4	-2.04462	-2.27237
FIGN	-4.11673	-2.16916	BSPRY	-2.73767	-4.32473
GPAT2	-2.35695	-2.98845	HYDIN	-2.31734	-2.25861
CLCA1	-3.54213	-2.87264	PAX1	2.597	2.459962
FXYD3	-2.18122	-2.3453	DLG2	3.181918	2.681872
OTC	-3.42591	-2.1234	SMIM17	-3.61519	-2.42362
DOC2B	-2.38589	-2.05822	CDKN2A	-3.04657	-4.28602
WDR86	2.023389	2.168281	TMEM125	-2.54586	-3.78701
TCP11	-3.28579	-2.23146	PCDHB14	2.463959	2.053336
POTED	2.112004	3.342583	KIF12	2.928755	2.355521
EFR3B	2.401565	2.037214	NCR1	2.584517	2.349407
GJB5	-2.28009	-3.28369	PAGE2	-3.36686	-2.42492
FSTL5	-3.91227	-3.33296	GPR110	-3.69825	-2.75823
KLF8	-2.50546	-3.39795	SYNPO2	3.980735	2.601894
TMEM119	3.509629	2.256734	MARVELD3	-3.29208	-3.9271
CDHR5	-2.95779	-2.19735	C5orf46	-2.76101	-2.9685
OVOL3	3.947104	2.045916	RHBG	-2.6235	-3.08807
OR4C11	2.205223	2.265899	JPH1	-5.70968	-3.5794
FRS3	4.643916	2.278968	SPRR3	-4.29344	-4.5551
SPINK2	-3.55611	-2.75492	EML5	2.471371	2.455401
TSSK3	-3.20599	-2.25785	INTS6	-2.67063	-2.24266
KCNV2	2.793343	2.257926			
ZBED6CL	-2.88975	-4.12526			
SIRPB2	-2.67235	-2.59034			

c

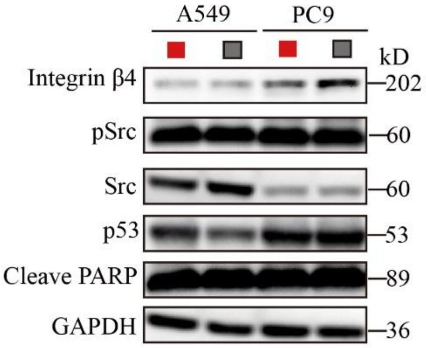

Fig. S7: Potential targets of circRABL2B in lung cancer cells. a, b Genes

displayed a more than twofold change in expression after circRABL2B knock-down in both A549 and PC9 are presented. **c** WB showing knock-down of YBX1 restored expressions of integrin β4, pSrc, p53, cleave PARP caused by circRABL2B overexpression. Two independent experiments were carried out with similar results.

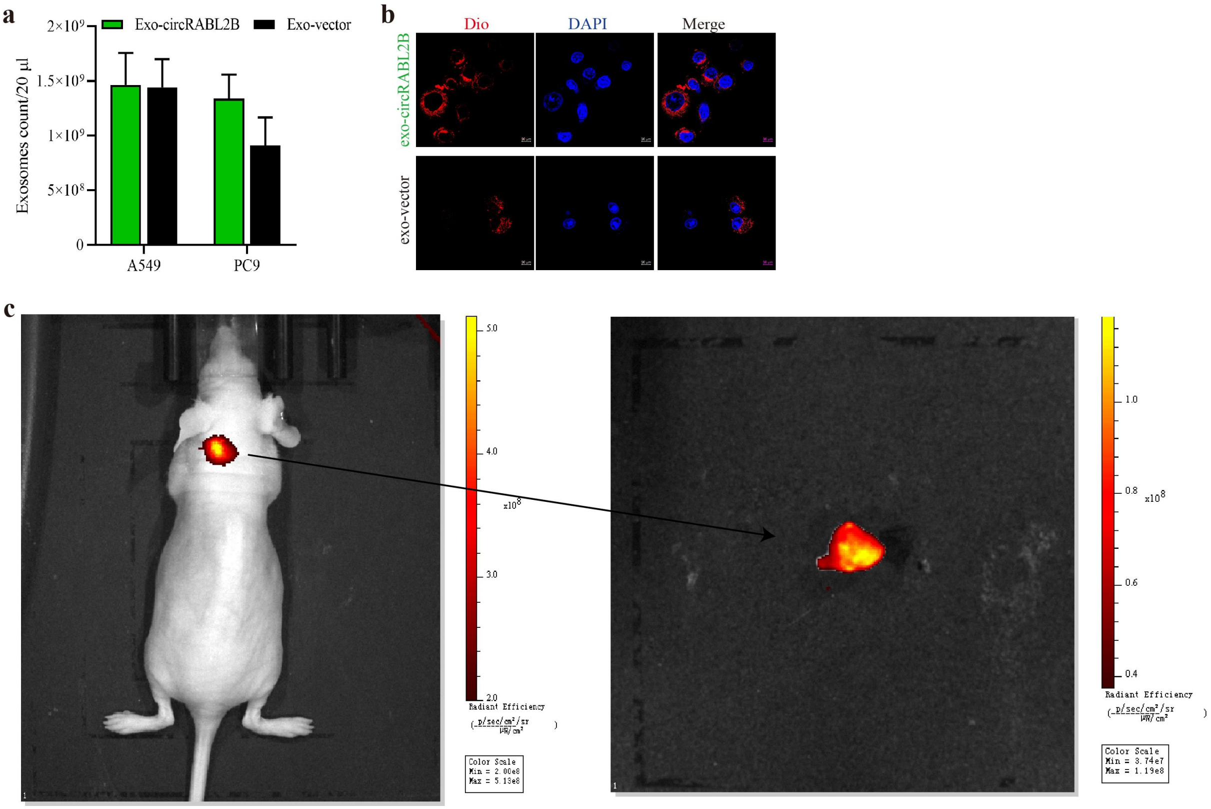

Fig. S8: Exosomes qualitative and quantitative analysis and tracking. a Quantitation of exosomes by ELISA. Data are presented as Mean ± S.E from 7-8 biologically replicates. *P* value is calculated by unpaired *t* test. **b** Cell image showing uptake exosomes *in vitro* using DiO Labeling Dye. **c** Live animal image showing uptake of exosomes *in vivo* using DiR Labeling Dye.

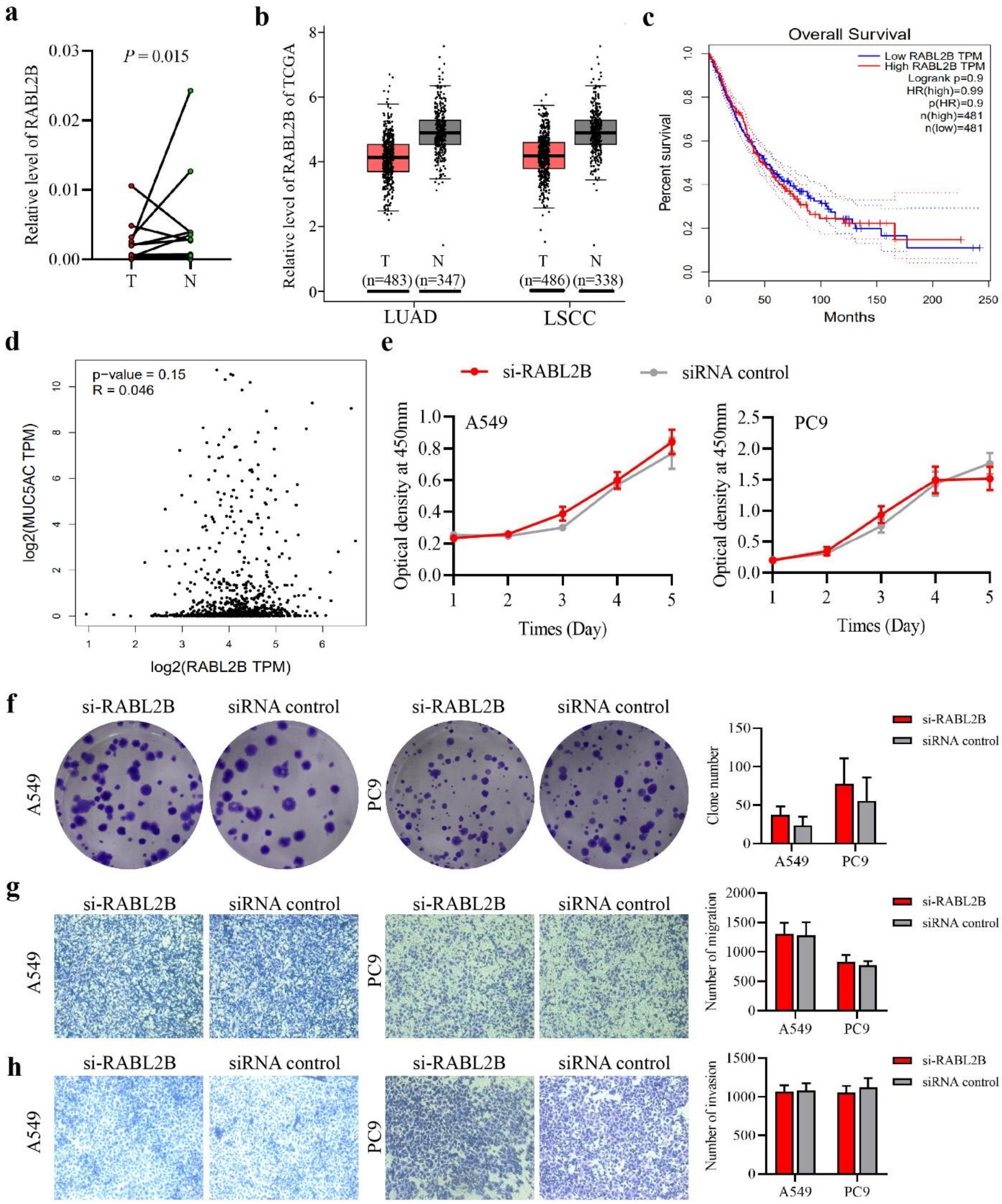

Fig. S9: Effect of the mother gene of circRABL2B on lung cancer. a, b The mRNA level of RABL2B in lung cancer tissues and adjacent normal tissues. **c** Survival analysis for RABL2B expression on lung cancer using the TCGA data. **d** Correlation analysis for MUC5AC and RABL2B using the TCGA data. **e-h** Assays for testing cell

proliferation (e), colony formation (f), migration (g) and invasion (h) in lung cancer cells with RABL2B knock-down (si-RABL2B) versus siRNA control. Data are presented as Mean ± S.D. from 3-6 biologically replicates for different assays. *P* values are calculated by paired *t* test in a, unpaired *t* test in f-h and Two-way ANOVA test in e.

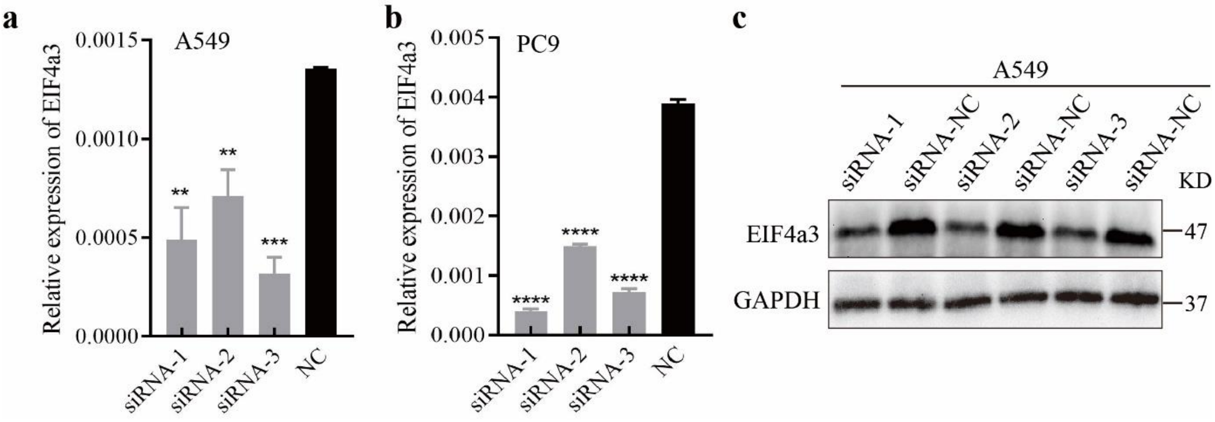

Fig. S10: Knockdown of EIF4a3 by siRNAs in lung cancer cells. a qPCR for testing EIF4a3 mRNA level after siRNAs treatment. **b** WB for testing EIF4a3 protein level after siRNAs treatment.
